# Ecology and allometry predict the evolution of avian developmental durations

**DOI:** 10.1038/s41467-020-16257-x

**Published:** 2020-05-14

**Authors:** Christopher R. Cooney, Catherine Sheard, Andrew D. Clark, Susan D. Healy, András Liker, Sally E. Street, Camille A. Troisi, Gavin H. Thomas, Tamás Székely, Nicola Hemmings, Alison E. Wright

**Affiliations:** 1 Department of Animal and Plant Sciences, University of Sheffield, Western Bank, Sheffield, S10 2TN UK; 2 School of Biology, University of St Andrews, St Andrews, KY16 9TH UK; 3 School of Earth Sciences, University of Bristol, Bristol, BS8 1TQ UK; 4 Department of Biology, Tufts University, 200 Boston Avenue, Medford, MA 02155 USA; 5 Department of Limnology, University of Pannonia, Pf. 1588201 Veszprém, Hungary; 6 MTA-PE Evolutionary Ecology Research Group, University of Pannonia, Pf. 158820 Veszpré, Hungary; 7 Department of Anthropology, Durham University, Durham, DH1 3LE UK; 8 School of Biological, Earth and Environmental Science, University College Cork, Cork, T23 N73K Ireland; 9 Department of Biology and Biochemistry, Milner Centre for Evolution, University of Bath, Bath, BA2 7AY UK; 10 Department of Evolutionary Zoology and Human Biology, University of Debrecen, Egyetem ter 1, H-4032 Debrecen, Hungary; 11 State Key Laboratory of Biocontrol and College of Ecology and Evolution, Sun Yat-sen University, Guangzhou, 510275 China; 12 Ministry of Education Key Laboratory for Biodiversity Sciences and Ecological Engineering, College of Life Sciences, Beijing Normal University, Beijing, 100875 China

**Keywords:** Behavioural ecology, Macroecology, Evolutionary developmental biology, Phylogenetics

## Abstract

The duration of the developmental period represents a fundamental axis of life-history variation, yet broad insights regarding the drivers of this diversity are currently lacking. Here, we test mechanistic and ecological explanations for the evolution of developmental duration using embryological data and information on incubation and fledging for 3096 avian species. Developmental phases associated primarily with growth are the longest and most variable, consistent with a role for allometric constraint in determining the duration of development. In addition, developmental durations retain a strong imprint of deep evolutionary history and body size differences among species explain less variation than previously thought. Finally, we reveal ecological correlates of developmental durations, including variables associated with the relative safety of the developmental environment and pressures of breeding phenology. Overall, our results provide broad-scale insight into the relative importance of mechanistic, ecological and evolutionary constraints in shaping the diversification of this key life-history trait.

## Abstract (web summary)

Developmental duration is a key life-history trait. Cooney et al. compile data on 3096 bird species to quantify the degree to which phylogenetic history, body size and ecological variables like predation risk or breeding phenology influence variation in developmental duration.

## Introduction

A fundamental goal in ecology and evolution is to explain the vast diversity of life-history strategies observed in nature^1–3^. The duration of the developmental period represents a fundamental axis of life-history variation^4^ and varies from days to several years among animal species. Attempts to explain variation in developmental duration across species typically fall into two broad categories^5^. A first set of hypotheses, focusing on the role of mechanistic constraints, predict that developmental periods vary among species largely as a result of negative allometric scaling between mass-specific growth (metabolic) rates and body size^6–8^. Growth is fuelled by metabolism, which scales negatively with body size, such that larger species have lower relative metabolic rates than smaller species, and thus take proportionally longer to develop^6–8^. If mechanistic constraints related to growth rate and body size during ontogeny represent an important rate-limiting step in offspring development, then phases of development associated with growth should be more variable across species and account for a greater proportion of total developmental time than non-growth periods.

A second set of hypotheses emphasise the role of ecology in generating interspecific differences in developmental durations. These ideas stem from classic life-history evolution theory^2,4,9^ and assume that the external context of the organism drives the evolutionary optimisation of growth rates (and hence developmental period), within the constraints imposed by size and other assumed trade-offs^5^. A range of extrinsic factors have been suggested to be important in driving the evolution of developmental duration, many of which relate to either (i) environmental or ecological limits to the resources available for reproduction or (ii) selection imposed by increased mortality of parents and/or offspring. Field studies focusing on one or a few species have provided critical insight into relationships between ecology, selection and variation in developmental periods^10–20^, but the restricted nature of these studies, combined with their often-conflicting results, have made broad conclusions difficult to draw.

Despite both sets of hypotheses being rooted in robust theoretical arguments, confidence in each is undermined by a lack of broad-scale empirical support and uncertainty exists regarding the relative importance of different factors for explaining broad-scale variation in developmental rates, particularly after accounting for phylogenetic effects^21^. Here we address this problem by conducting a global-scale phylogenetic comparative analysis of avian developmental periods and other life-history traits. Birds are particularly suited for such analyses, because accurate information on the duration of major avian developmental periods (incubation and fledging) is available for many species, as well as for many relevant aspects of species’ biology, ecology and distribution. In addition, detailed data on embryonic developmental stages are available for several taxonomically diverse bird species, permitting the integration of large-scale comparative analyses with fine-scale investigation into differences in species’ developmental rates. To quantify broad-scale variation in overall developmental period length across birds, we follow previous studies (e.g., refs. ^22–25^) and use the sum of incubation and fledging periods, thereby capturing variation in both pre-natal and post-natal development rates. Furthermore, by combining data on incubation and fledging duration, we are able to define a second variable, which we refer to as the incubation fraction. This variable captures differences among species in the balance between prenatal (incubation) and postnatal (fledging) development periods, and is calculated as the duration of the incubation period divided by the total developmental duration (incubation + fledging). This is useful, because in birds, as in other animals, the proportion of prenatal to postnatal development varies among species, raising important questions regarding the factors explaining these differences (e.g., developmental mode, predation risk, etc.)^26,27^. However, although the importance of such factors have been examined in some taxa^10–20^, their importance is rarely tested at broad phylogenetic scales.

To test key mechanistic and ecological explanations for the evolutionary diversification of developmental durations in birds, we collect data from two different sources. First, we extract standardised estimates of developmental timepoints from a taxonomically diverse sample of 20 bird species with existing information on embryonic development. We predict that if mechanistic constraints related to growth rates play an important role in determining avian developmental durations, then developmental phases associated primarily with growth should be longer and more variable across species than earlier phases concerned mostly with cell differentiation and body plan formation. Second, we compile information on incubation and fledging durations for a total of 3096 bird species from 176 families and 39 orders, and combine this in a phylogenetic comparative framework with comprehensive data for a suite of variables that have previously been linked to avian developmental durations^10–20^. Specifically, we test variables related to species’ body mass, life-history, parental care, nesting behaviour, ecology, ambient climate and biogeography. This two-scale approach—combining detailed observations of embryonic development with a broader comparative dataset—allows us to (i) to identify the phase(s) of avian development contributing most to interspecific differences in developmental duration, (ii) investigate the strength of phylogenetic signal in trait values, and (iii) directly test and compare the relative importance of multiple potential underlying factors for determining developmental period length. We use this approach to quantify the relative roles of mechanistic constraints and species’ ecology in shaping the evolution of avian developmental durations at a global scale.

## Results and discussion

### Growth stages of development are the longest and most variable

**Fig. 1:**
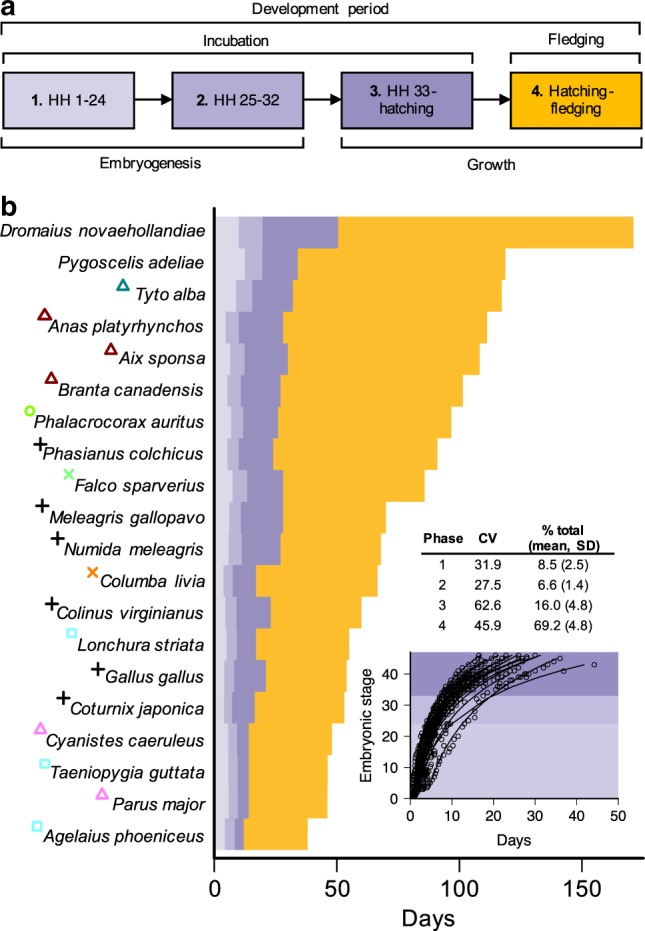
The duration of avian developmental phases. **a** Schematic illustrating four distinct phases of avian ontogeny. Phase 1 and phase 2 corresponding to Hamburger–Hamilton stages (HH) 1–24 and 25–32, respectively, represent embryonic developmental stages primarily associated with embryogenesis (i.e., non-growth). In contrast, phases 3 (HH33 to hatching) and 4 (post-hatching fledging period) correspond to developmental periods consisting largely of growth. **b** Stacked bar chart showing time intervals associated with phases 1 to 4 for 20 bird species with available information on the timing of embryonic developmental stages, with species are ordered by total developmental duration. Coloured symbols next to species names correspond to the major taxonomic groups identified in Fig. 2. Inset graph shows the staging data and fitted curves used to estimate the time points separating phases 1–3. Inset table reports the coefficient of variation (CV) and percentage of total developmental period length (% total) accounted for by each of the four phases. Source data are provided as a Source Data file.

To test the prediction that growth periods represent the longest and most variable phases of offspring development, we conducted a fine-scale analysis of developmental rates in a taxonomically diverse set of species (*n* = 20) with existing information on the timing of key developmental stages (Supplementary Fig. 1 and Supplementary Table 1). Specifically, we examined four distinct phases in avian ontogeny spanning both pre-hatching (incubation) and post-hatching (fledging) periods (Fig. 1a). Phases 1 and 2, defined on the basis of Hamburger–Hamilton (HH) stages 1–24 and 25–32, respectively, correspond to periods of chick embryogenesis. These early stages of prenatal development consist primarily of cell differentiation and embryo formation rather than absolute growth^28^, and we therefore consider these as “non-growth” phases. In contrast, phases 3 and 4, corresponding to HH33 to hatching and hatching through to fledging, are primarily concerned with periods of prenatal (phase 3) and postnatal (phase 4) growth of existing structures (see Supplementary Methods for extended justification of these developmental phases). We used this framework to investigate variation in the duration and partitioning of avian offspring development.

We found that the durations of developmental stages associated with embryogenesis (phases 1 and 2) account for only a small proportion of the variance in overall developmental duration across species (Fig. 1b). At these early stages of development, all bird embryos—regardless of species identity and eventual adult body size—are of comparatively similar size and therefore expected to have approximately similar growth rates^7^. In contrast, the durations of growth phases (phases 3 and 4) are longer and more variable than non-growth phases and account for a far greater proportion of the variance in developmental duration among species (Fig. 1b). The longer duration of growth phases relative to embryogenesis phases is consistent with the well-characterised phenomenon of declining growth rates over ontogeny, caused by decreases in the ratio of energy acquisition to energy loss as developing organisms increase in size^5^. Furthermore, greater variance in the duration of growth phases relative to non-growth phases (as indicated by coefficient of variation scores; Fig. 1b) is also consistent with greater size-related effects on the later stages of development. As development progresses, offspring of different species become increasingly different in size and therefore exhibit far greater disparity in relative growth rates compared with earlier stages of development.

### Developmental durations are phylogenetically conserved

**Fig. 2:**
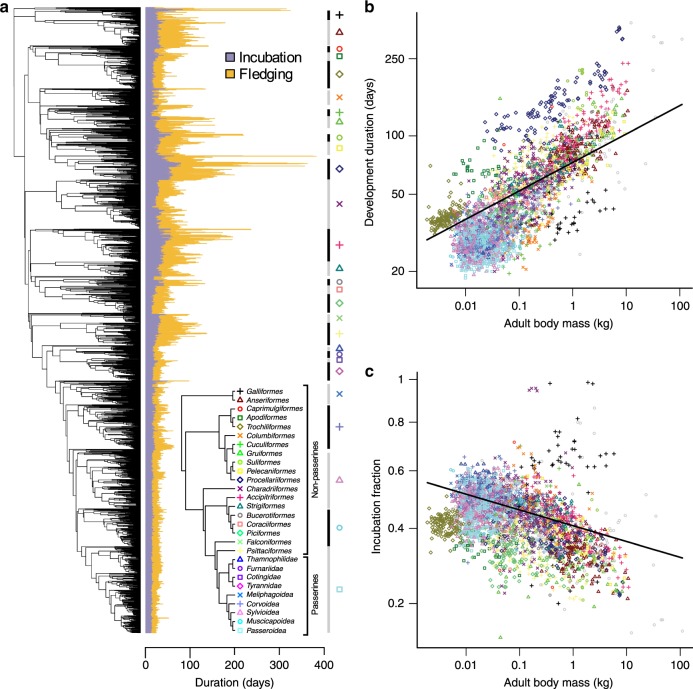
The diversity, phylogenetic distribution and allometry of development periods in birds. **a** The phylogenetic distribution of incubation, fledging and total development (incubation + fledging) period across 3096 species of birds. Inset tree schematic indicates the relationships among major taxonomic groups (>20 spp.) and provides a key for the plotting symbols used throughout the figure. **b**, **c** Allometric relationships of (log-transformed) development period length (**b**) and (square root-transformed) incubation fraction (**c**) with (log-transformed) adult body mass. Lines indicate the regression slopes estimated by phylogenetic regression. Source data are provided as a Source Data file.

Our observation that the developmental phases associated primarily with growth are longer and more variable than earlier non-growth phases predicts that body size should explain a significant amount of variation in developmental durations due to metabolic scaling rules. To test this idea more broadly, we collected data on developmental durations (incubation and fledging periods) for 3096 bird species covering the breadth of the avian phylogeny (Fig. 2a and Supplementary Fig. 2). In our dataset, overall developmental durations ranged from ~20 days in some passerine species (e.g., *Volatinia jacarina*) to >350 days in some seabird lineages (e.g., *Diomedea*). Likewise, estimates of the proportion of development allocated to incubation relative to fledging (i.e., the incubation fraction) also varied markedly across species, ranging from ~0.15 (e.g., *Struthio camelus*) to >0.95 in certain landfowl and shorebird species (e.g., *Megapodius pritchardii* and *Synthliboramphus wumizusume*).

Before addressing relationships with body size, we first quantified the extent of phylogenetic signal in avian developmental durations. Fitting Pagel’s *λ* model^29^ to our dataset, we found that developmental variables exhibited strong phylogenetic signal, with *λ* values [95% confidence interval (95% CI)] of 0.93 [0.91, 0.94] and 0.86 [0.83, 0.89] for developmental duration and incubation fraction, respectively. This reflects a pervasive pattern in our dataset, that species within clades tend to exhibit similar developmental durations and incubation fractions (Fig. 2a), such that on average closely related species have more similar trait values than more distantly related species.

### Body size explains less variation than previously thought

Against this backdrop of evolutionary conservatism, we used phylogenetic regression^30^ and variance partitioning techniques^31^ to test the relationships between body size and avian developmental duration while jointly estimating phylogenetic effects, and to compare the contributions of predictor variables (body size) and variance components (phylogenetic effects) to the overall fit of the model. Using this approach, as predicted we found that overall developmental duration is positively related to body size across bird species (Fig. 2b). Furthermore, for incubation fraction we found that the offspring of larger-bodied species have proportionally shorter incubation periods relative to fledging periods (Fig. 2c), presumably reflecting energetic and/or ecological constraints associated with laying and/or developing in larger eggs. In both cases, we used adult body mass values as our index of body size across species, which we consider to represent a useful albeit imperfect proxy for offspring size at the end of development. However, we note that results were similar when we use an alternative proxy for offspring size (initial egg mass) (Supplementary Table 2). Variance partitioning revealed that the partial *R*^2^ values associated with these phylogenetically-adjusted allometric relationships were 0.22 and 0.05 for developmental duration and incubation fraction, respectively. In contrast, the partial *R*^2^ values associated with the phylogenetic (covariance) components of each model were far greater: 0.79 and 0.59, respectively.

The significant relationships we observe between body size and developmental durations are in line with our predictions based on embryological data and provide broad empirical support for the role of size-related constraints in determining both the duration and partitioning of avian developmental periods^6–8^. However, after accounting for phylogenetic effects, we found that the importance of body size for explaining variation in developmental durations across birds was surprisingly low, particularly considering that early tests implied that as much as 85% of interspecific variation in incubation period could be explained by body size effects^32–34^. In contrast, our comparatively low estimates for the variance explained by body size (5–22%) support the conclusion that, although important, allometric constraints play a more minor role in determining the length and partitioning of avian developmental periods than once thought^21^. Instead, our quantitative estimates indicate that a greater proportion of the variance in avian developmental durations is attributable to phylogenetic history rather than body size. This finding is apparent in the observation that species within clades typically share similar developmental duration values that are largely unrelated to variation in body size both within and between clades (Fig. 2 and Supplementary Fig. 3).

### Ecology predicts broad-scale variation in development periods

**Fig. 3:**
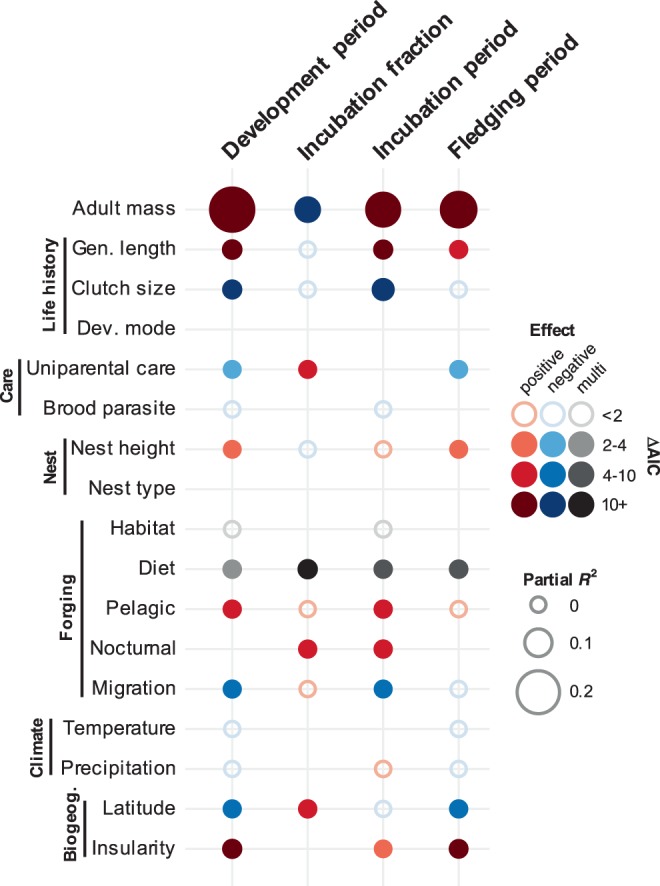
Predictors of the duration and partitioning of developmental period lengths in birds. Phylogenetically controlled multi-predictor models of development period, incubation fraction, incubation period and fledging period. Unfilled circles indicate factors that were significant as single predictors but not significant in a multi-predictor model. Gaps indicate factors that were not significant (ΔAIC < 2) as single predictors and were therefore not included in the multi-predictor model. Red and blue points indicate predictors with positive and negative effects, respectively. Predictors with grey points (e.g., Diet) represent categorical variables with >2 (‘multi’) levels. ΔAIC values indicate the change in model support when the focal predictor was dropped from the model, with larger ΔAIC values indicating greater support for the importance of a predictor. Sample sizes (number of species) for the models were 1665, 1685, 1935 and 1665 for development period, incubation fraction, incubation period and fledging period, respectively.

The existence of substantial mass-independent differences in developmental periods among bird lineages is intriguing, as it raises questions regarding the relative importance of mechanistic versus ecological constraints in generating interspecific diversity in avian developmental periods. However, such questions have yet to be addressed at broad scales^21^. The idea that ecology plays an important role in driving the evolution of developmental periods is rooted in classical life-history optimisation theory^2,4,9^. To test the relative importance of ecology in explaining broad-scale variation in developmental periods, we studied the individual and combined effects of 16 variables related to behavioural, ecological, environmental, and life-history variation across species (plus body size) that have previously been linked to patterns of selection acting on avian developmental periods^10–20^ (Fig. 3). Specifically, we used phylogenetic generalised least squares (PGLS) regression with optimised Pagel’s lambda^29^ to test for relationships between ecological traits and developmental duration that are predicted by adaptive hypotheses. This approach, which is based on an underlying Brownian motion (BM) model, is suitable for testing for evolutionary associations between variables across species while controlling for the degree of phylogenetic non-independence in the data^35^. As such, this represents an appropriate framework within with to address our hypotheses as we are able to robustly test for correlations between ecological traits and developmental durations that are predicted by adaptive hypotheses, while simultaneously estimating and correcting for the degree of phylogenetic effects^31,35^. Furthermore, we performed a model comparison analysis and found that the lambda model greatly outperformed alternative candidate models [strict BM, Ornstein-Uhlenbeck (OU)] for the phylogenetic covariance structure of the residuals of our models and therefore represents the most appropriate statistical model with which to analyse our dataset.

Our analyses revealed several important correlates of variation in avian developmental durations. First, after testing each predictor separately (see Supplementary Figs. 4–7), we found strong relationships between several variables and developmental duration and incubation fraction across species (Supplementary Table 2). By combining all significant single predictors in multi-predictor models, we were then able to identify sets of important predictors with unique effects that are independent of phylogeny. We found that, in addition to being larger, species with longer overall developmental durations tend to be longer lived, with smaller clutches, biparental care, elevated nest heights, vertebrate-eating/scavenging dietary niches, and pelagic foraging ecologies (Fig. 3 and Supplementary Table 3). These species also tend to be non-migratory and have more equatorial and insular breeding-range distributions. For incubation fraction, in addition to the negative relationship with body size, we found that species with proportionally longer incubation periods tend to have uniparental parental care, are typically insectivorous and nocturnal, and have more polar breeding-range distributions (Fig. 3 and Supplementary Table 4). In both cases, broadly similar effects were found using initial egg mass as an alternative proxy for body size (Supplementary Fig. 8 and Supplementary Tables 5 and 6). Partial *R*^2^ values for these models indicated that, after controlling for phylogenetic and body size effects, the unique effects of ‘ecological’ variables included in multi-predictor models accounted for ~12% and ~4% of the variance in developmental duration and incubation fraction, respectively (Table 1). Interestingly, the magnitude of these effects were similar to those associated with body size (Table 1), implying that ecological and allometric effects (as measured here) explain roughly equivalent proportions of variation in developmental durations among bird species. Nonetheless, the variance associated with phylogenetic components indicated that phylogenetic effects remained a dominant source of variation in these models (Table 1). In total, these models incorporating body size, ecological, and phylogenetic effects accounted for 62–93% of the variation in developmental durations across species.

**Table 1:** *R*^2^ values for model components explaining variation in avian developmental durations.

Model component	Development period	Incubation fraction	Incubation period	Fledging period
Body size	0.18	0.05	0.12	0.13
Ecology	0.12	0.04	0.11	0.06
Phylogeny	0.62	0.43	0.70	0.52
Full model	0.91	0.62	0.93	0.84

Partial *R*^2^ (individual components) and *R*^2^ (full model) values are derived from phylogenetic multi-predictor models of development period, incubation fraction, incubation period and fledging period. Sample sizes and predictor sets are the same as those given in Fig. 3.

These results have several important implications. Most notably, they show that behavioural and ecological variables among species are significant predictors of variation in developmental durations across species, consistent with an important role for ecology in driving the evolution of avian developmental durations^4^. In particular, three main ‘ecological syndromes’ appear to be associated with variation in developmental durations. First, longer developmental durations are generally associated with factors that presumably increase the safety of the developmental environment from predation threat or other mortality risks, such as nesting in relatively inaccessible sites (nest height), on islands (insularity), or having more than one parent to provide for and protect the offspring (biparental care). The idea that nesting in safe places may relax selection for rapid development is consistent with work by Remeš and Martin^14^, who found nestling growth rates to be positively associated with predation rates across passerines. Second, factors linked to phenological effects, such as breeding at temperate latitudes, insectivory, and migratory ecology, tend to be associated with shorter developmental durations. In species where reproductive success is driven largely by an individual’s ability to coincide their reproduction with peak seasonal food availability^4,36,37^, the need to operate within a tight timeframe to avoid phenological mismatch is likely to select for rapid development^38^. Third, several of the patterns we observe are also consistent with the importance of trade-offs between reproduction and survival for determining variation in avian developmental strategies. For example, shorter developmental periods in species with short lifespans and large clutches are consistent with selection for ‘fast’ life-histories and greater investment in reproduction (independent of body size)^39,40^, whereas longer developmental periods among species with vertebrate hunting/scavenging diets are potentially explained by selection for slower development to mitigate costs associated with limited and/or unpredictable food availability^41,42^.

Furthermore, by considering predictors of incubation and fledging period separately, our results provide further insight into the patterns of selection generating underlying divergence in overall developmental duration and pre- versus postnatal allocation (Fig. 3 and Supplementary Tables 7–10). For instance, our finding that nocturnal species have larger incubation fractions than diurnal species is seemingly driven by nocturnal species having relatively long incubation periods rather than particularly short fledging stages. This makes sense if lower daytime parental activity disproportionally reduces nest predation risk during the incubation period relative to the fledging period^19^, thus relaxing selection for rapid development inside the egg. Similarly, the longer developmental periods of pelagic species are largely driven by relatively long incubation periods, which may be a consequence of selection for advanced development at hatching^15^ or lower rates of egg predation due to inaccessible breeding locations^40^.

In contrast, our results show that species with uniparental care tend to have overall shorter developmental durations (and greater incubation fractions) largely because of reduced fledging durations. This is consistent with predictions for evolutionary associations between single parent care and short post-hatching offspring development periods^4,43^, but the direction of causality remains unclear. On the one hand, uniparental care may generate selection for rapid post-hatching offspring development to reduce the burden of care, but on the other hand short post-hatching periods may facilitate desertion by one of the parents (typically the male), implying a reversal in the direction of cause and effect^44^.

Finally, our results challenge several assumptions regarding relationships between developmental durations and other factors at broad scales. In particular, ambient climate is predicted to shape broad-scale patterns of developmental rates in birds via its effect on egg temperature and parental behaviour^21^. However, after controlling for the effect of other factors, we found no evidence that variation in environmental conditions (temperature and precipitation) was related to developmental duration across species. This finding supports the view that offspring are to a large extent buffered from variation in ambient environmental conditions by parental adaptations such as nest design, incubation efficiency and provisioning rate^17,45,46^.

Surprisingly, we also found no significant relationships with developmental mode (precocial, semi-precocial, altricial) or nest type (cavity, closed, open, mixed). This is despite strong expectations for significant associations^26,47–49^ and seemingly large differences between groups in the raw data (see Supplementary Figs. 4–7). We attribute these negative results to the effect of correcting for phylogenetic non-independence among species in our models. Variation in developmental mode and nest type are phylogenetically conserved across the avian phylogeny^50^. Power to detect significant relationships with traits that have independently evolved only a few times is limited and so their effects cannot be disentangled from underlying patterns of shared evolutionary history and/or ecology^31^. Greater clarity on whether factors such as developmental mode and nesting behaviour directly influence the evolution of developmental durations or are simply associated at broad scales via phylogenetically conserved constraints will likely come from integrating data on equivalent traits from other groups (e.g., all vertebrates) to generate sufficient independent phylogenetic replication to conclusively test these relationships.

### Conclusions

Overall, our study reveals key drivers of developmental durations across the breadth of the avian phylogeny, providing broad, quantitative insight into the relative importance of mechanistic constraints and ecologically mediated selection in explaining variation in key life-history traits. Furthermore, our results highlight the pervasive impact of phylogenetic history in shaping variation in species’ developmental durations. The close association between developmental duration, species’ traits and phylogeny implies a strong signal of evolutionary conservatism, both in terms of species’ developmental durations and the combinations of factors (‘syndromes’) that co-evolve with them, echoing the conclusions from other large-scale phylogenetic analyses of avian life-history traits^40,50^. Although birds provide sufficient evolutionary replication to investigate the importance of many factors, phylogenetic constraints and evolutionary conservatism makes it difficult to tease apart the effects of other, less labile, traits. Thus, a potentially fruitful avenue of future research would be to address these questions over even broader phylogenetic scales to better address the effects of body size, species’ traits and phylogenetic constraints (e.g., mutation rates) on the evolutionary diversification of developmental durations.

## Methods

### Data

We collected information on the timing of embryonic development for 20 species using data available in the primary literature (see Supplementary Table 1). For each species with data, we extracted information on the time taken for embryos to reach sequential stages of the HH^28^ scale, which represents a standard approach for describing and comparing rates of embryonic development across bird species^51^. To ensure consistent measurements across species, one of us (NH) re-staged embryo development using data provided in the original publication. In cases where an alternative staging approach was used, we re-staged embryo development according to the Hamburger and Hamilton^28^ scale using detailed descriptions and photographs provided in the original publication. In cases where a range of time points were reported for reaching a given stage, we used the average.

We collected information on prenatal (incubation) and postnatal (fledging) period lengths (days) for 3096 bird species from ref. ^52^ and major ornithological reference works^53^. Following ref. ^52^, we define incubation period as the time (in days) between when the egg is laid and when it hatches, and fledging period as the time taken (in days after hatching) for offspring to be capable of flight (or for some species, leaving the nest). These variables have been used extensively in the comparative avian life-history literature and represent standardised measurements of avian developmental periods that are broadly comparable across all bird species^52^. Although we acknowledge that in some bird lineages individuals continue to grow after fledging, we argue that in most cases post-fledging growth accounts for a relatively minor proportion of offspring development and as such the combined duration of incubation plus fledging periods represents an informative metric of the total development time.

To improve data quality, we removed clear outliers that must reflect measurement error (i.e., incubation lengths <8 or >90 days; *n* = 6). In addition, we also assessed the extent of within-species variability in development period estimates (where available) relative to the extent of variation across all species by calculating repeatability (i.e., intra-class correlation) coefficients^54^. Our dataset contained an average of 1.54 (range 1–9) measurements per species for incubation period and 1.47 (range 1–9) measurements per species for fledging period. Based on this data, estimated repeatability coefficients were 0.984 (95% CI = [0.982, 0.985]) for incubation period and 0.944 (95% CI = [0.939, 0.949]) for fledging period, implying low variability in estimates of developmental periods within species relative to variation between species. We therefore calculated mean values of incubation and fledging period per species (when multiple values were available) and from this calculated variables capturing total developmental duration (incubation + fledging) and incubation fraction [incubation/(incubation + fledging)].

Data on adult body mass (g), initial egg mass (g), generation length (days), clutch size, developmental mode (precocial, semi-precocial, altricial), parental care (uniparental, biparental), brood parasitism (parasite, non-parasite), minimum nest height (m), nest type (cavity, closed, open, mixed), habitat (forest dependency: high, medium, low, none), diet (omnivore, fruit/nectar, invertebrate, plant/seed, vertebrate/fish/scavenger), foraging (pelagic, non-pelagic), nocturnality (nocturnal, diurnal) and migration (sedentary, migratory) were extracted from standard avian trait databases^52,55,56^ or scored directly from the literature (primarily ref. ^53^). Geographical variables, including temperature, precipitation, latitudinal midpoint and insularity (continental, insular), were based on maps of species’ breeding distributions from http://www.datazone.birdlife.org (Version 9) combined with global climate^57^ and landmass datasets^58^. Further details of data compilation methods are given in the Supplementary Methods.

### Phylogeny

Our analyses are based on the taxonomy and phylogenies of ref. ^59^, which currently represent the only available ‘complete’ species-level phylogenetic hypothesis for all birds. To provide a phylogenetic framework for the species in our dataset (*n* = 3096), we downloaded 1000 ‘full’ trees (those containing all 9993 species) based on the ‘Hackett’ backbone from http://www.birdtree.org, which we then pruned to leave only the species represented in our dataset. We then used this tree distribution to generate a maximum clade credibility tree, which provided the phylogenetic framework for our analyses.

### Categorising avian development into stages

We categorised avian development into four discrete phases spanning both prenatal (embryonic; based on the descriptions of ref. ^28^) and postnatal (fledging) periods (Fig. 1a), and calculated the time taken for individuals to reach the end of each phase. Specifically, we estimated the time required for embryos to reach HH24 (phase 1), HH33 (phase 2), to hatch (phase 3) and finally to fledge (phase 4). To estimate the time points associated with reaching HH24 and HH33, we fitted curves of the form:

1 $${{y}}={\mathrm{e}}^{a+bx}$$

using the R function ‘nls’ to describe the relationship between embryonic stage (*x*) and time (*y*) (Supplementary Fig. 1). This allowed us to accurately infer time points associated with HH24 and HH33, even when such data were not explicitly reported in the original publication. Data on the later time points (hatching and fledging) were extracted either from the relevant staging paper directly or else imported from our broader comparative dataset.

### Model comparison analyses

We identified a number of hypotheses that outline a role for allometry and ecology in generating interspecific differences in developmental durations. These hypotheses predict relationships between variables (i.e., body size, ecological traits) and developmental duration, which we test for using PGLS regression. PGLS generates phylogenetically corrected slope and intercept estimates for relationships between variables, which can be used to test predictions for the nature of the evolutionary association between traits derived from different hypotheses^35^. Within this framework, one of the most commonly used models for the covariance structure of the residuals of relationships between traits is Pagel’s lambda model. This model is useful as it provides a quantitative estimate of phylogenetic signal (based on deviations from a BM model), which can be used to flexibly adjust such tests according to the level of phylogenetic non-independence in the data^31,35^. However, other models that make different assumptions about the evolutionary process (e.g., strict BM, OU) can be used to model the expected covariance structure of residuals from these relationships. Therefore, to assess the suitability of the lambda model relative to other candidate models for analysing our dataset, we conducted a model comparison analysis.

To do this, we ran three different versions of each PGLS model tested, with each version assuming a different evolutionary model for the covariance structure of the residuals (strict BM, OU, lambda). We assessed support for the different model types on the basis of log-likelihood values and ΔAIC scores, the latter of which provides a measure of relative model support. All models were fit using the ‘phylolm’ function in the R package ‘phylolm’ (ver. 2.6)^30^ and R code used to conduct these analyses is available via GitHub (see Data Availability section). In the case of the OU model, we further note that the PGLS-based model fitting approach used here (using phylolm) is analogous to other implementations of the OU model (e.g. those in OUCH or SLOUCH)^60^, in which one trait (i.e. the predictor) defines the optima to which another trait (i.e. the response) is assumed to evolve.

We found that in all cases BM and OU models were greatly disfavoured relative to the lambda model for describing the covariance structure of our PGLS models. Specifically, across all models average ΔAIC scores were 3441 (range 808–5144) and 2674 (range 591–4289) for the BM and OU models, respectively, where ΔAIC scores > 20 are usually taken to indicate essentially no empirical support for the candidate model^61^. Correspondingly, in all cases, these scores translated into AIC weights of ~0 for the BM and OU models, and ~1 for the optimised lambda model, indicating strong statistical support for the use of the lambda model in this context. Full results for these trait model comparison analyses can be found in Supplementary Table 11. Therefore, on the basis of these results, and in conjunction with the arguments outlined above, we conclude that PGLS with optimised lambda is a suitable framework—both conceptually and in practice—with which to conduct our analyses.

### Allometric analyses

We tested the relationship between adult body size and developmental period variables while jointly estimating phylogenetic effects using the approach outlined above (i.e., PGLS with optimised lambda as a measure of the degree of phylogenetic signal; R code available via GitHub, see ‘Data Availability section’). For each response variable, we also fit a model in which intercepts were estimated separately for major taxonomic groups (>20 spp.), to generate mass-adjusted estimates of relative developmental durations (Supplementary Fig. 3).

### Multi-predictor models

We used the same PGLS regression approach described above to test the relationship between predictor variables and variation in our developmental variables. First, we fitted individual (i.e., single) predictor models using all available data for each predictor. We then combined all individually important predictors into a multi-predictor model. We note that due to missing data in predictor variables, sample sizes for multi-predictor models were reduced relative to the size of the full dataset. However, the subset of species included in these analyses were broadly distributed across the avian phylogeny (Supplementary Fig. 2) and distributed among taxonomic groups in approximately the same proportions as in the full dataset (Spearman’s *r* = 0.95–0.96, *P* < 0.001 in all cases), implying that the sample of species included in our multi-predictor analyses are representative of our larger dataset and of avian diversity more generally.

In all cases, predictors were considered to be important if model support values dropped by >2 units (i.e., ΔAIC > 2) when the predictor was dropped from the model while holding lambda values constant^61^, with larger ΔAIC values indicating greater statistical support for the importance of a predictor. We checked for evidence of multi-collinearity among predictors in our multi-predictor models using variance inflation factors (VIFs) and found no evidence of severe (VIF > 10) or even moderate (VIF > 5) multi-collinearity in any of our models (median VIF = 1.80; range = 1.01 – 4.13). *R*^2^ values for full models (including phylogenetic effects) and partial-*R*^2^ values associated with predictors were calculated using the ‘R2.lik’ function in the R package ‘rr2’^31^. R code used to run these analyses is available via GitHub (see ‘Data Availability section’).

### Reporting summary

Further information on research design is available in the Nature Research Reporting Summary linked to this article.

## Supplementary information

Supplementary Information

Reporting Summary

## Data Availability

Data analysed in this study are available as a Source Data file, which corresponds to the data plotted in Figs. 1 and 2, and Supplementary Figs. 3–7.

## Source data

Source Data

## Code availability

Full details on R packages and functions used are listed throughout and R code used to run our analyses, including the model comparison analysis involving BM and OU models, is available at https://github.com/christophercooney/Avian-developmental-durations.
