# Supplementary material for: Ecology and allometry predict the evolution of avian developmental durations
Source: Nat Commun. 2020 May 14;11:2383. doi: 10.1038/s41467-020-16257-x (PMC7224302; doi:10.1038/s41467-020-16257-x)
Supplement: Supplementary file 1 — Supplementary Information [file 41467_2020_16257_MOESM1_ESM.pdf]

Supplementary Information for

Ecology and allometry predict the evolution of avian developmental durations

Christopher R. Cooney*, Catherine Sheard, Andrew Clark, Susan Healy, András Liker, Sally E. Street, Gavin H. Thomas, Camille A. Troisi, Tamás Székely, Nicola Hemmings* & Alison E. Wright*

***Authors for correspondence:** c.cooney@sheffield.ac.uk, n.hemmings@sheffield.ac.uk, a.e.wright@sheffield.ac.uk

Supplementary Methods

Extended justification for avian developmental phases. According to Hamburger & Hamilton [ref. (1)], stages 1-24 of embryo development (our 'Phase 1'; Fig. 1A) are described exclusively in terms of embryogenesis – specifically the formation and organisation of the fundamental body plan. During this time (up to incubation day 4 in the chicken), changes in the embryo are characterised primarily by the number of somites, and then, once somites become difficult to see due to the development of the mesoderm, the development of limb-buds, visceral arches, and other externally visible structures. Similarly, stages 25-32 (incubation day 4-8 in the chicken; our 'Phase 2') are also characterised by rapid developmental changes in the wings, legs, and visceral arches, and can therefore also be considered as part of embryogenesis, as the differentiation of body structures (e.g. toes, mandible, etc.) is still ongoing.

In contrast, from stage 33 onwards, chick development is described primarily in terms of growth, rather than embryogenesis. Specifically, between stages 33-38 (incubation day 8-12 in the chicken), Hamburger and Hamilton describe changes in feather germs and eyelids to distinguish stages, both of which are already present in the developing embryo. Furthermore, from stage 38 (day 12 in the chicken) onwards, Hamburger and Hamilton explicitly state that no new structures are formed, and that chick development primarily comprises the growth of structures that already exist. Thus, from stage 38 onwards, Hamburger and Hamilton exclusively use measurements of beak and toe length (i.e. growth) to distinguish stages. We decided to include stage 33-38 into this 'growth' phase (our 'Phase 3') because although Hamburger and Hamilton were not exclusively using growth measurements to differentiate stages at this point, they were still using descriptions of growth based on existing structures only. Thus, we consider chick development from stage 33 to hatching (our 'Phase 3'), and from hatching to fledging (our 'Phase 4'), to constitute growth, in contrast to stages 1-32, which we consider to represent embryogenesis.

Predictor variables. Data on mean adult body mass (g), egg mass (g), clutch size, diet (omnivore , fruit/nectar, invertebrate, plant/seed, vertebrate/fish/scavenger), foraging (pelagic, non-pelagic) and nocturnality (nocturnal, diurnal) were extracted directly from refs. (2) and (3). We used the literature [primarily refs. (4) and (5)] to assign species to broad categories capturing variation in developmental mode (precocial, semi-precocial, altricial), parental care (uniparental, biparental), brood parasitism (parasite, non-parasite), nest type (cavity, closed, open, mixed). Nest height (m) was recorded as the (minimum) distance between the base of the egg cup and the ground for a given species reported in the literature. We extracted information on generation length (days), habitat (forest dependency: high, medium, low, none) and migration (sedentary, migratory) from http://www.datazone.birdlife.org following the approaches described in ref. (6). Briefly, regarding species' habitat classifications, in the

BirdLife dataset species are assigned to one of four broad habitat categories, depending on whether they ‘do not normally occur in forests’, or exhibit ‘low’, ‘medium’ or ‘high’ levels of forest dependency. Similarly, BirdLife categorise species as ‘not a migrant’, ‘nomadic’, ‘altitudinal migrant’ or ‘full migrant’. We converted this classification system into a binary variable capturing broad differences in species’ migratory tendencies, categorising each species as ‘non-migratory’ or ‘migratory’ (nomadic, altitudinal migrant or full migrant).

Variables relating to species’ geographical distributions are based on bird breeding range maps provided by BirdLife International and NatureServe (version 9; http://www.datazone.birdlife.org), rasterised to 1° resolution. Following ref. (7), we calculated average range-wide temperature and precipitation values for the warmest quarter (bio10 and bio18), extracted from the WorldClim2 database (8), and we calculated species mean (absolute) breeding-range latitude values directly from grid cell occurrences.

Finally, insularity was determined by comparing species range maps to a dataset of global landmasses (http://www.soest.hawaii.edu/pwessel/gshhg/; v2.3.6), and we defined insular species as those with >95% of their range occurring on islands as defined by ref. (9). Prior to analysis, incubation fraction was square-transformed, and the following variables were log-transformed: incubation, fledging and total developmental duration, adult body mass, generation length, clutch size and nest height. The full dataset is provided as Source Data.

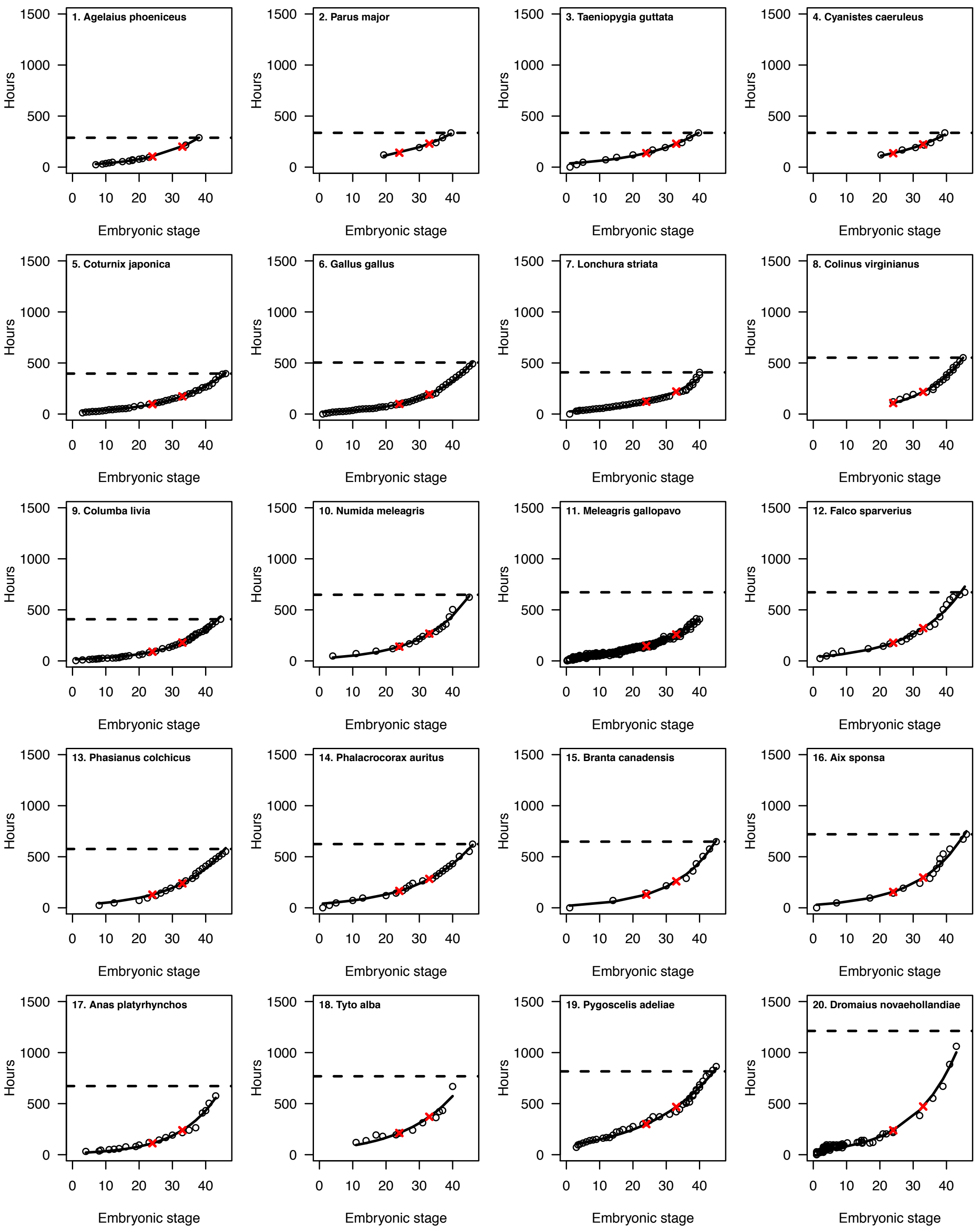

Supplementary Fig. 1. Individual embryonic development curves for 20 bird species. Points are observed data and fitted lines come from fitting an equation of the form y = exp(a + b * x). Red crosses indicate the estimated time at which species reach embryonic stage 24 and 33, respectively. Dotted line indicates the hatching time, as reported from the relevant literature.

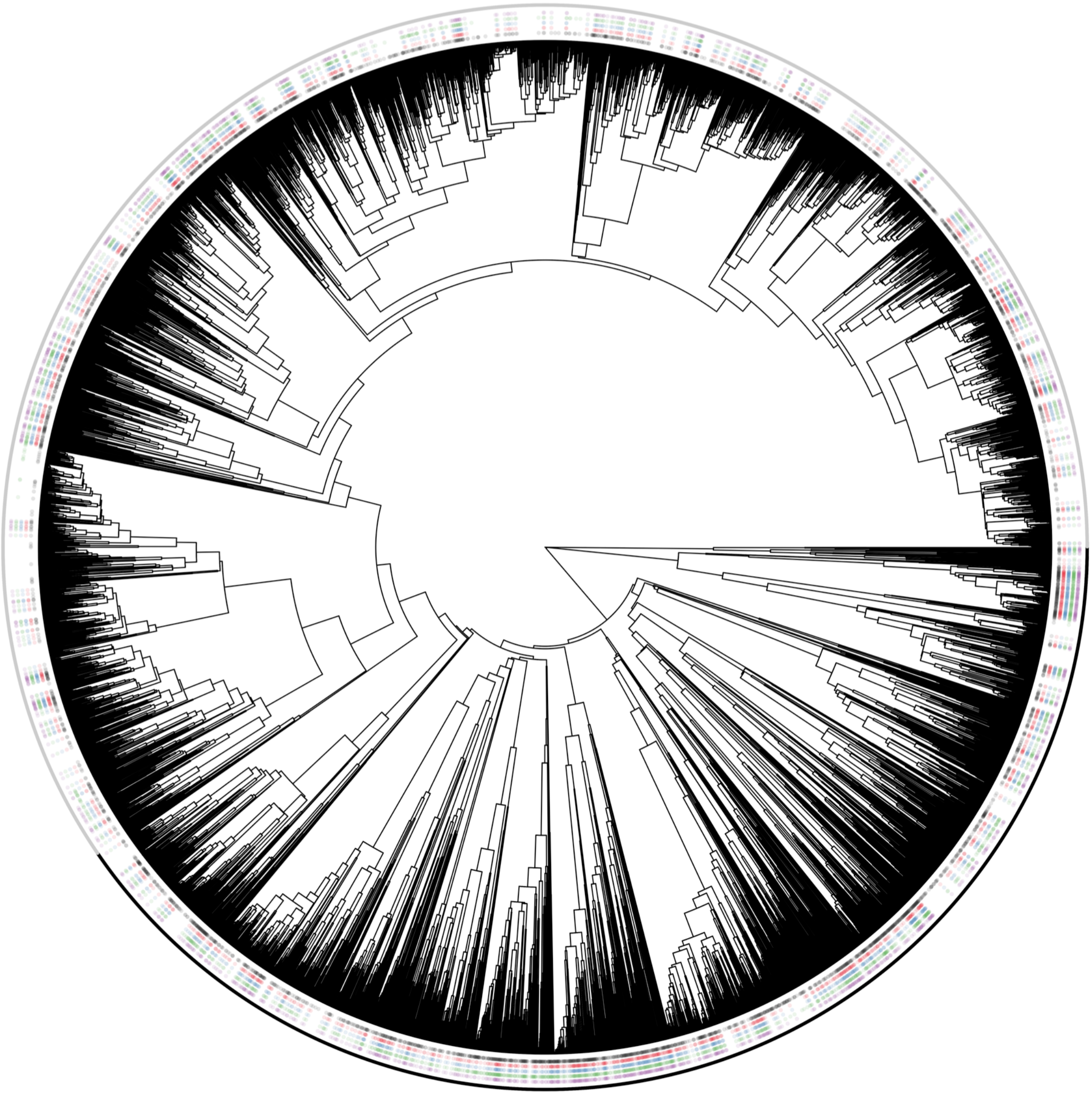

Supplementary Fig. 2. The phylogenetic distribution of species sampled in this study. The plot shows a representative 'complete' phylogeny from birdtree.org (containing 9,993 species) based on the 'Hackett' backbone with dots at the tips indicating the phylogenetic position of species included in different analyses. The inner ring (black dots) corresponds to species comprising the full dataset (3,096 species), with subsequent rings indicating species included in datasets underlying the four multi-predictor models shown in Fig. 3 [red = development period (1,665 spp.); blue = incubation fraction (1,685 species); green = incubation period (1,935 species); purple = fledging period (1665 species)]. The outer rings correspond to non-passerine (black) and passerine (grey) lineages.

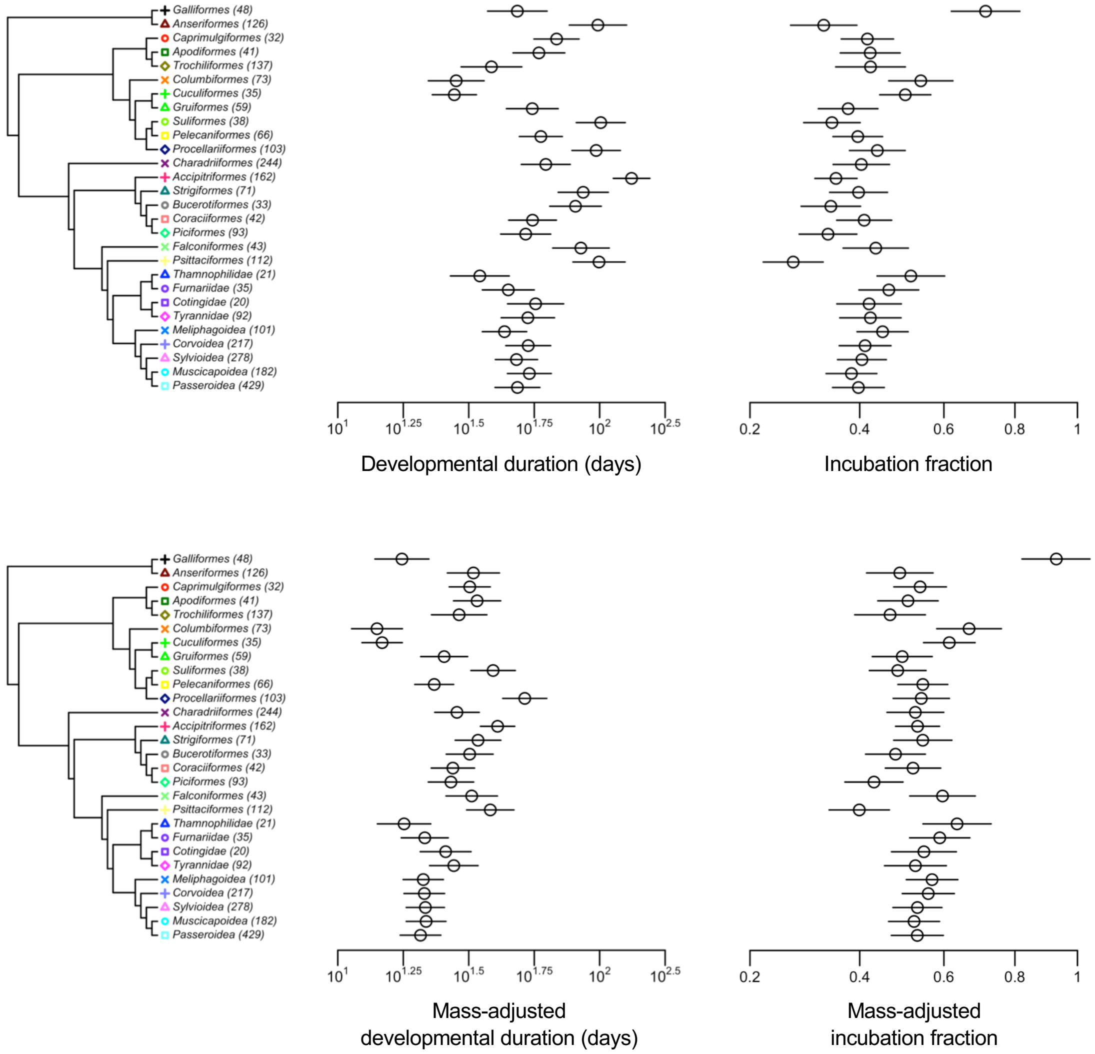

Supplementary Fig. 3. Variation in developmental durations of major avian clades. Relative developmental duration and incubation fraction values represent the *y*-intercepts from a model of the form *y* = *a* + *b* (log mass) in which major avian clades (>20 spp.) were permitted to have unique intercepts (but parallel slopes). Horizontal lines indicate standard errors of model parameter estimates. Sample sizes for the number of species in each clade are shown in parentheses.

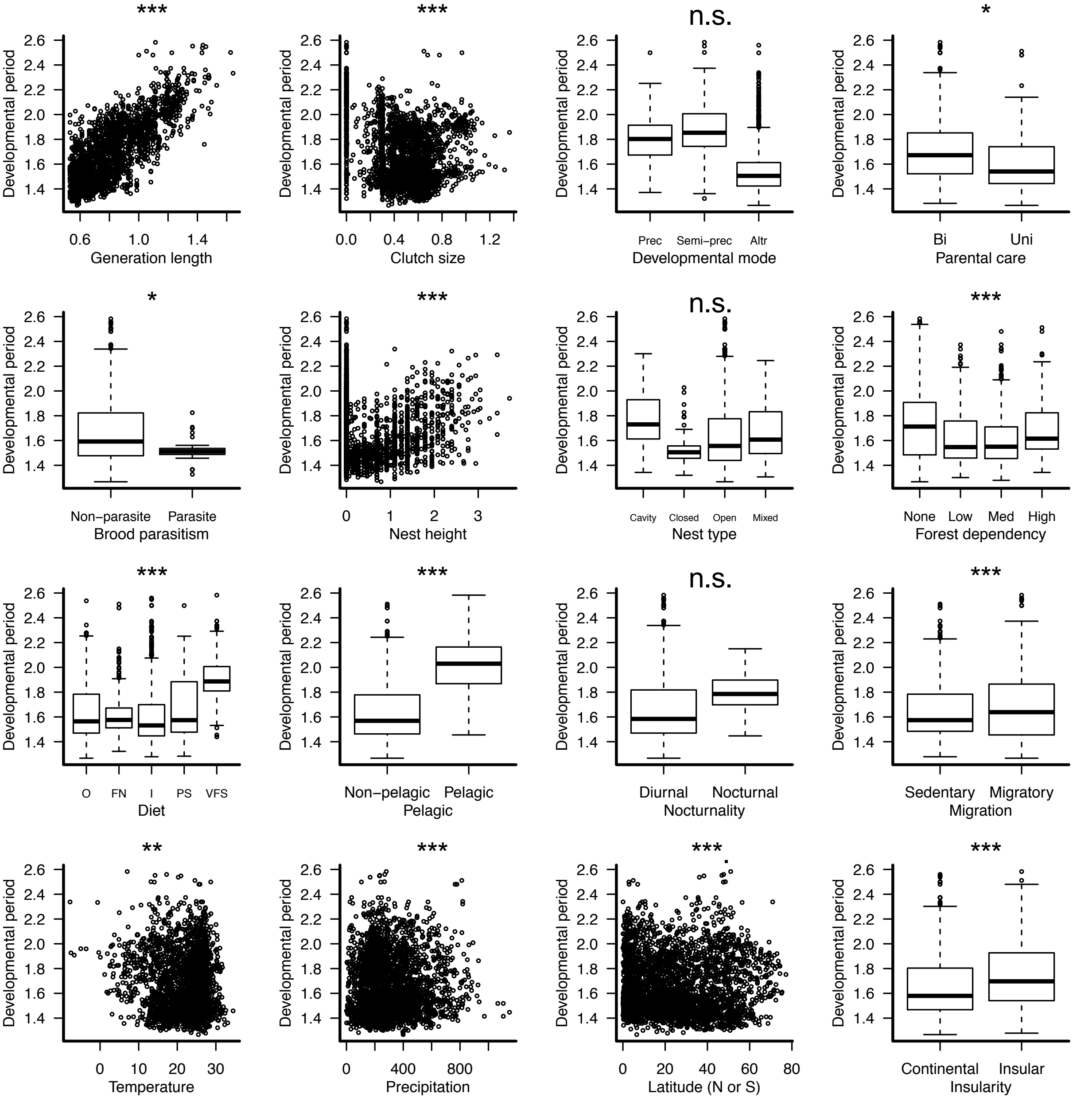

Supplementary Fig. 4. Relationships between (log_{10}-transformed) total developmental period length and individual predictor variables. Box and whisker plots for categorical predictors show the median (centre line) and interquartile range (box) of the data, the range of data which is within 1.5 times the interquartile range of the box (whiskers), and the position of outliers (points) that lie beyond this range. Relationships were tested using two-sided PGLS regression (see Methods). Asterisks denote significant univariate relationships (see Supplementary Table 2). *, $P < 0.05$; **, $P < 0.01$; ***, $P < 0.001$; n.s., not significant ($P > 0.05$). Source data are provided as a Source Data file.

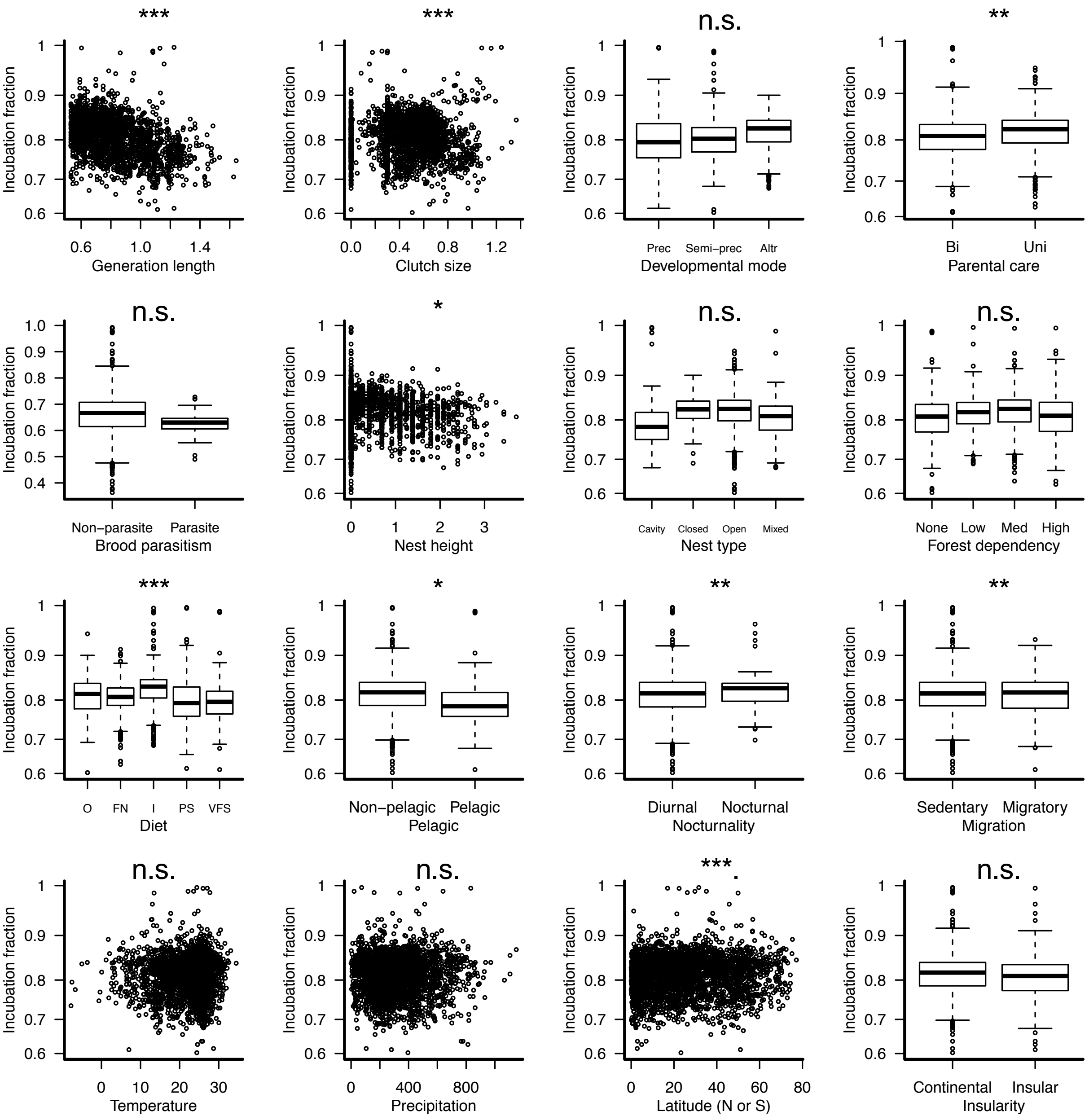

Supplementary Fig. 5. Relationships between (square root-transformed) incubation fraction and individual predictor variables. Box and whisker plots for categorical predictors show the median (centre line) and interquartile range (box) of the data, the range of data which is within 1.5 times the interquartile range of the box (whiskers), and the position of outliers (points) that lie beyond this range. Relationships were tested using two-sided PGLS regression (see Methods). Asterisks denote significant univariate relationships (see Supplementary Table 2). *, $P < 0.05$; **, $P < 0.01$; ***, $P < 0.001$; n.s., not significant ($P > 0.05$). Source data are provided as a Source Data file.

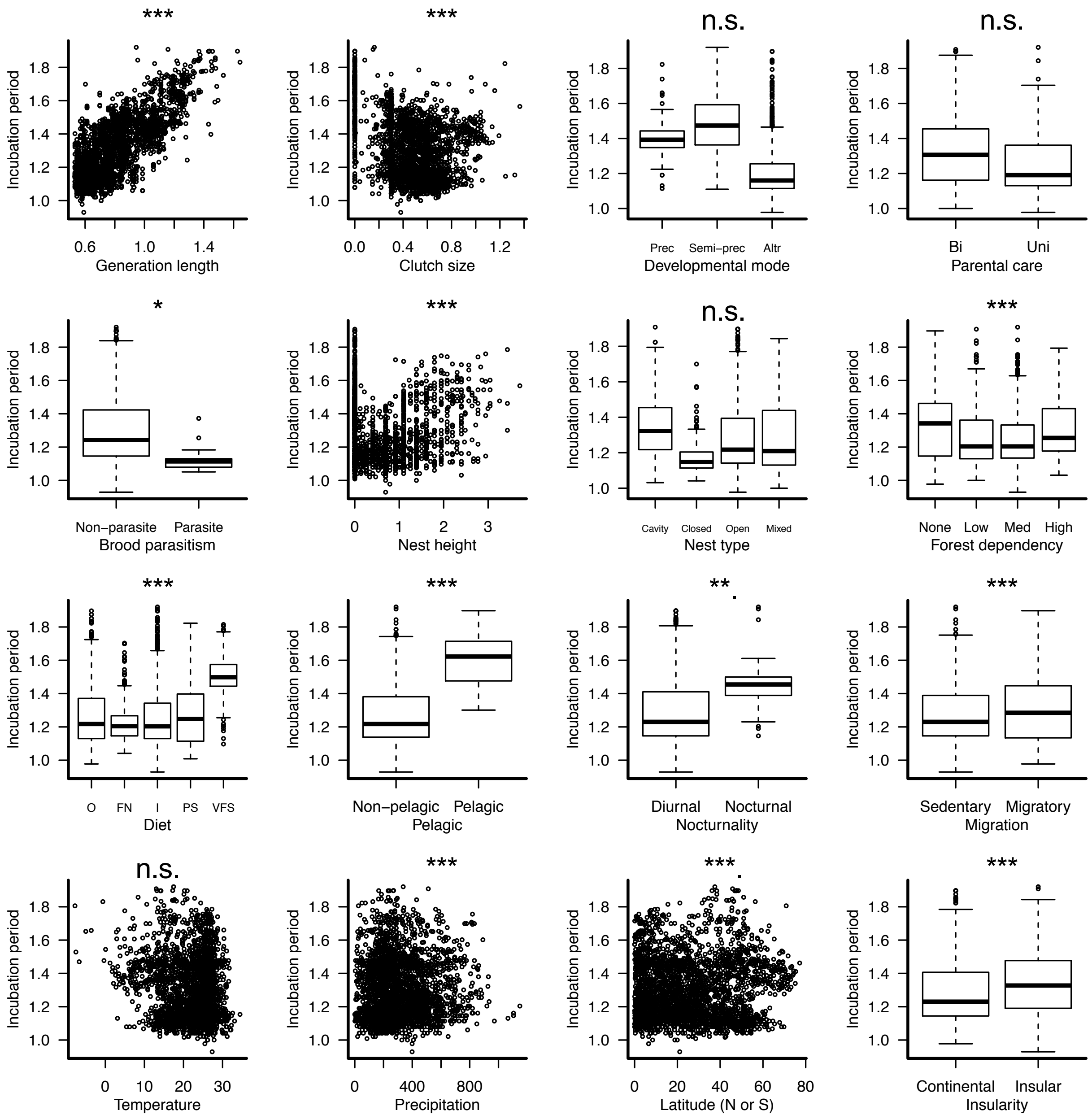

Supplementary Fig. 6. Relationships between (log$_{10}$-transformed) incubation period length and individual predictor variables. Box and whisker plots for categorical predictors show the median (centre line) and interquartile range (box) of the data, the range of data which is within 1.5 times the interquartile range of the box (whiskers), and the position of outliers (points) that lie beyond this range. Relationships were tested using two-sided PGLS regression (see Methods). Asterisks denote significant univariate relationships (see Supplementary Table 2). *, $P < 0.05$; **, $P < 0.01$; ***, $P < 0.001$; n.s., not significant ($P > 0.05$). Source data are provided as a Source Data file.

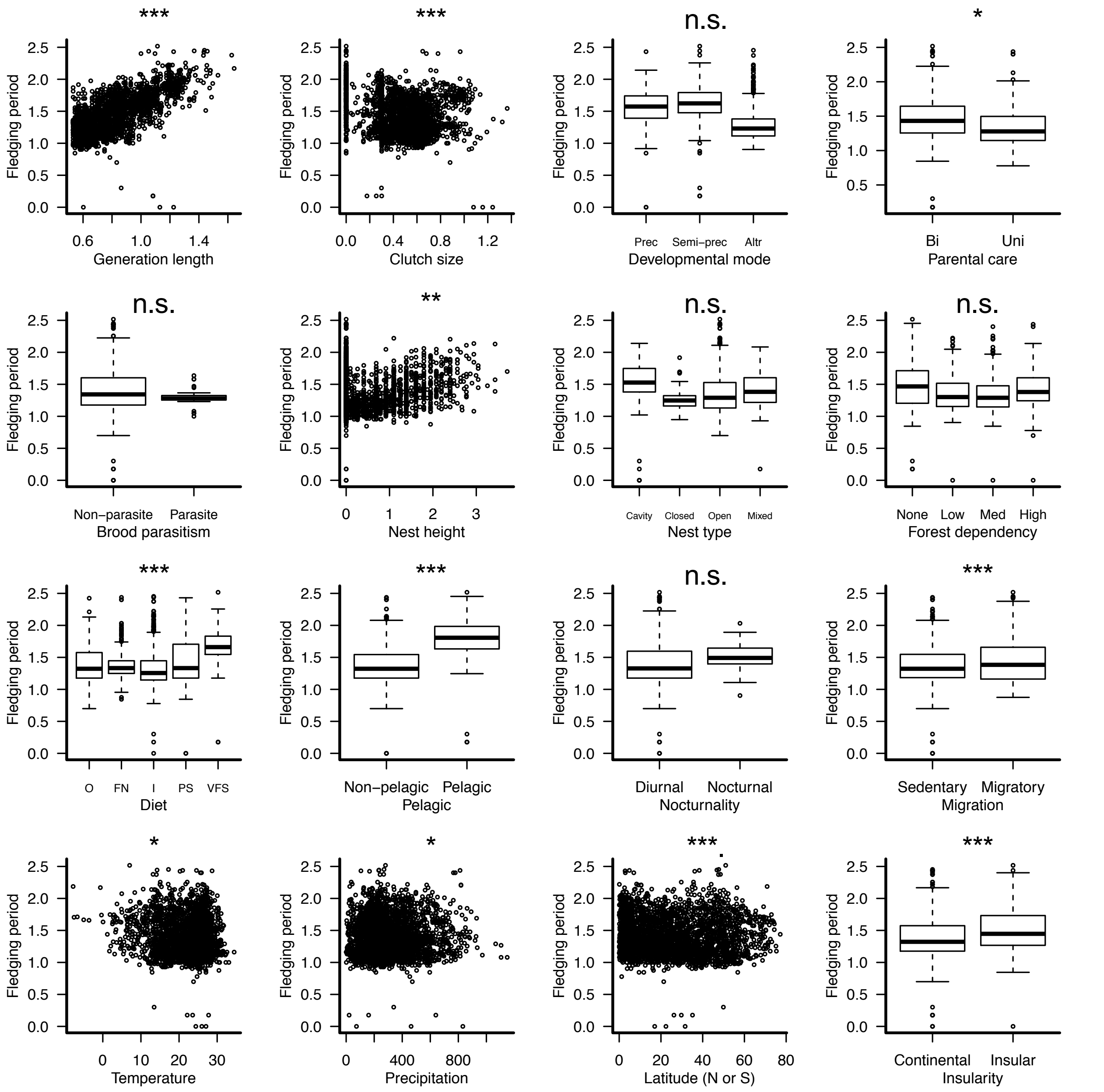

Supplementary Fig. 7. Relationships between (log_{10}-transformed) fledging period length and individual predictor variables. Box and whisker plots for categorical predictors show the median (centre line) and interquartile range (box) of the data, the range of data which is within 1.5 times the interquartile range of the box (whiskers), and the position of outliers (points) that lie beyond this range. Relationships were tested using two-sided PGLS regression (see Methods). Asterisks denote significant univariate relationships (see Supplementary Table 2). *, $P < 0.05$; **, $P < 0.01$; ***, $P < 0.001$; n.s., not significant ($P > 0.05$). Source data are provided as a Source Data file.

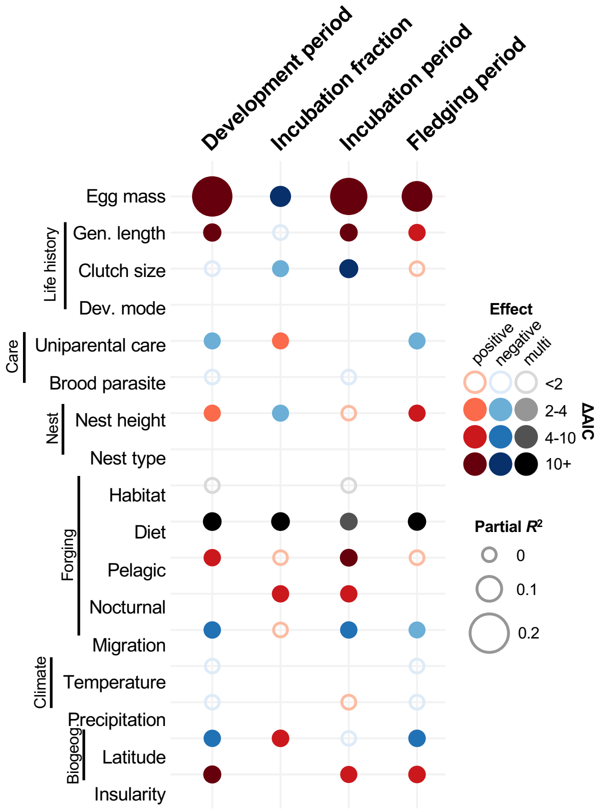

Supplementary Fig. 8. Predictors of the duration and partitioning of developmental period lengths in birds using egg mass as a proxy for size. Phylogenetically-controlled multi-predictor models of development period, incubation fraction, incubation period and fledging period. Unfilled circles indicate factors that were significant as single predictors but not significant in a multi-predictor model. Gaps indicate factors that were not significant (ΔAIC < 2) as single predictors and were therefore not included in the multi-predictor model. Red and blue points indicate predictors with positive and negative effects, respectively. Factors with filled grey points (e.g. Diet) represent categorical variables with >2 ('multi') levels. ΔAIC values indicate the change in model support when the focal predictor was dropped from the model, with larger ΔAIC values indicating greater support for the importance of a predictor. Sample sizes (number of species) for the models were 1448, 1468, 1673, 1448 for development period, incubation fraction, incubation period and fledging period, respectively.

Supplementary Table 1. Sources of embryological development data.

Species	Common name	Reference
Agelaius phoeniceus	Red-winged blackbird	(10)
Aix sponsa	Wood duck	(11)
Anas platyrhynchos	Mallard	(12)
Branta canadensis	Canada goose	(13)
Colinus virginianus	Northern bobwhite	(14)
Columba livia	Rock dove	(15)
Coturnix japonica	Japanese quail	(16)
Dromaius novaehollandiae	Emu	(17)
Falco sparverius	American kestrel	(18)
Gallus gallus	Chicken	(1)
Lonchura striata	Society finch	(19)
Meleagris gallopavo	Turkey	(20)
Numida meleagris	Guineafowl	(21)
Cyanistes caeruleus	Blue tit	(22)
Parus major	Great tit	(22)
Phalacrocorax auritus	Double-crested cormorant	(23)
Phasianus colchicus	Pheasant	(24)
Pygoscelis adeliae	Adelie penguin	(25)
Taeniopygia guttata	Zebra finch	(22)
Tyto alba	Barn owl	(26)

Supplementary Table 2. Single predictor models of avian developmental periods.

		Development period						Incubation fraction						Incubation period						Fledging period					
Term	N	Estimate	SE	T	P	ΔAIC	Partial R^2	Estimate	SE	T	P	ΔAIC	Partial R^2	Estimate	SE	T	P	ΔAIC	Partial R^2	Estimate	SE	T	P	ΔAIC	Partial R^2
(Intercept)	3096	1.423	0.034	41.724	0.000	783.347	0.224	0.756	0.019	39.836	0.000	170.250	0.054	1.179	0.027	43.555	0.000	560.463	0.166	1.018	0.056	18.073	0.000	488.188	0.146
Adult body mass		0.147	0.005	29.889	0.000			-0.039	0.003	-13.305	0.000			0.093	0.004	24.827	0.000			0.183	0.008	23.039	0.000		
(Intercept)	2600	1.504	0.034	44.008	0.000	673.644	0.229	0.720	0.020	36.028	0.000	92.236	0.036	1.214	0.025	47.984	0.000	644.191	0.220	1.141	0.059	19.307	0.000	347.914	0.126
Egg mass		0.207	0.007	27.766	0.000			-0.045	0.005	-9.792	0.000			0.142	0.005	27.074	0.000			0.242	0.012	19.346	0.000		
(Intercept)	2858	1.575	0.041	38.517	0.000	197.269	0.067	0.719	0.021	34.822	0.000	48.762	0.018	1.283	0.031	41.722	0.000	138.967	0.048	1.224	0.064	18.982	0.000	112.805	0.039
Generation length		0.289	0.020	14.361	0.000			-0.079	0.011	-7.154	0.000			0.176	0.015	12.017	0.000			0.340	0.031	10.819	0.000		
(Intercept)	2979	1.894	0.039	48.693	0.000	144.615	0.048	0.639	0.019	33.464	0.000	10.365	0.004	1.485	0.028	52.690	0.000	150.563	0.050	1.599	0.060	26.537	0.000	81.309	0.028
Clutch size		-0.142	0.012	-12.255	0.000			0.023	0.007	3.519	0.000			-0.103	0.008	-12.507	0.000			-0.165	0.018	-9.188	0.000		
(Intercept)	1765	1.829	0.043	42.627	0.000	-3.999	0.000	0.641	0.020	31.910	0.000	-0.382	0.002	1.425	0.030	46.984	0.000	0.981	0.003	1.537	0.067	22.853	0.000	-3.703	0.000
Dev. mode [semi-precocial]		0.000	0.012	-0.027	0.978			0.010	0.007	1.454	0.146			0.016	0.007	2.089	0.037			-0.006	0.019	-0.303	0.762		
Dev. mode [altricial]		0.000	0.015	-0.009	0.993			0.003	0.009	0.385	0.700			0.011	0.009	1.128	0.260			0.001	0.024	0.045	0.964		
(Intercept)	2623	1.829	0.041	44.999	0.000	3.381	0.002	0.631	0.019	33.715	0.000	5.860	0.003	1.416	0.030	47.069	0.000	-1.995	0.000	1.586	0.060	26.252	0.000	4.690	0.003
Parental care [uniparental]		-0.015	0.006	-2.320	0.020			0.010	0.004	2.805	0.005			0.000	0.004	0.067	0.946			-0.025	0.010	-2.587	0.010		
(Intercept)	3096	1.817	0.040	45.769	0.000	2.374	0.001	0.652	0.019	34.909	0.000	-1.987	0.000	1.429	0.029	48.858	0.000	3.899	0.002	1.510	0.060	25.022	0.000	0.167	0.001
Brood parasitism [parasitic]		-0.057	0.027	-2.091	0.037			0.002	0.015	0.116	0.908			-0.048	0.020	-2.429	0.015			-0.061	0.042	-1.472	0.141		
(Intercept)	2193	1.809	0.041	44.499	0.000	11.402	0.006	0.655	0.019	34.629	0.000	2.312	0.002	1.424	0.030	48.021	0.000	9.247	0.005	1.496	0.062	24.130	0.000	8.153	0.005
Nest height		0.011	0.003	3.665	0.000			-0.004	0.002	-2.077	0.038			0.007	0.002	3.356	0.001			0.015	0.005	3.189	0.001		
(Intercept)	2493	1.801	0.041	44.363	0.000	-0.402	0.002	0.665	0.019	34.539	0.000	-5.406	0.000	1.429	0.031	46.061	0.000	-1.905	0.002	1.462	0.062	23.460	0.000	-3.265	0.001
Nest type [closed]		0.011	0.009	1.192	0.234			-0.002	0.005	-0.381	0.703			0.011	0.007	1.519	0.129			0.019	0.015	1.291	0.197		
Nest type [open]		-0.004	0.007	-0.575	0.565			0.001	0.004	0.291	0.771			0.000	0.005	0.052	0.958			0.004	0.011	0.332	0.740		
Nest type [mixed]		-0.006	0.006	-1.031	0.302			0.000	0.003	-0.046	0.963			-0.001	0.004	-0.275	0.783			-0.001	0.009	-0.149	0.882		
(Intercept)	3061	1.812	0.040	45.658	0.000	9.431	0.005	0.651	0.019	34.754	0.000	-3.417	0.001	1.423	0.029	48.791	0.000	18.144	0.008	1.506	0.061	24.867	0.000	0.494	0.002
Forest dep. [low]		0.000	0.004	-0.028	0.978			0.002	0.002	0.972	0.331			0.001	0.003	0.535	0.593			-0.002	0.006	-0.286	0.775		
Forest dep. [med]		0.004	0.004	1.038	0.299			0.003	0.002	1.445	0.149			0.007	0.003	2.450	0.014			0.001	0.006	0.139	0.889		
Forest dep. [high]		0.019	0.005	3.587	0.000			0.001	0.003	0.256	0.798			0.018	0.004	4.704	0.000			0.017	0.008	2.110	0.035		
(Intercept)	3096	1.819	0.039	46.460	0.000	47.294	0.018	0.651	0.018	35.442	0.000	32.340	0.013	1.430	0.029	49.061	0.000	20.295	0.009	1.513	0.059	25.426	0.000	43.355	0.016
Diet [fruitnect]		0.015	0.007	2.135	0.033			-0.010	0.004	-2.424	0.015			0.002	0.005	0.477	0.634			0.024	0.011	2.204	0.028		
Diet [invert]		-0.019	0.005	-4.171	0.000			0.011	0.003	3.941	0.000			-0.007	0.003	-2.017	0.044			-0.031	0.007	-4.412	0.000		
Diet [plantseed]		-0.004	0.006	-0.726	0.468			-0.003	0.003	-0.949	0.343			-0.008	0.004	-1.848	0.065			-0.002	0.009	-0.236	0.813		
Diet [vertfishscav]		0.024	0.007	3.461	0.001			-0.006	0.004	-1.478	0.140			0.018	0.005	3.545	0.000			0.029	0.011	2.703	0.007		
(Intercept)	3096	1.815	0.039	46.273	0.000	46.862	0.016	0.652	0.019	34.921	0.000	3.411	0.002	1.427	0.029	49.576	0.000	42.728	0.014	1.507	0.060	25.119	0.000	26.063	0.009
Pelagic [pelagic]		0.076	0.011	7.016	0.000			-0.015	0.007	-2.326	0.020			0.052	0.008	6.710	0.000			0.088	0.017	5.308	0.000		
(Intercept)	3096	1.815	0.040	45.148	0.000	-1.931	0.000	0.643	0.019	34.259	0.000	7.883	0.003	1.415	0.030	47.897	0.000	7.541	0.003	1.520	0.061	24.914	0.000	-0.583	0.000
Nocturnality [nocturnal]		0.013	0.051	0.263	0.792			0.075	0.024	3.145	0.002			0.115	0.037	3.090	0.002			-0.091	0.077	-1.190	0.234		
(Intercept)	3061	1.822	0.039	46.148	0.000	71.102	0.024	0.651	0.019	34.723	0.000	7.928	0.003	1.432	0.029	49.217	0.000	59.143	0.020	1.515	0.060	25.090	0.000	38.435	0.013
Migration [migratory]		-0.026	0.003	-8.598	0.000			0.006	0.002	3.152	0.002			-0.017	0.002	-7.856	0.000			-0.030	0.005	-6.378	0.000		
(Intercept)	3033	1.795	0.040	44.397	0.000	7.440	0.003	0.659	0.019	34.533	0.000	0.842	0.001	1.419	0.030	47.593	0.000	1.713	0.001	1.487	0.061	24.200	0.000	2.175	0.001
Temperature		0.001	0.000	3.074	0.002			0.000	0.000	-1.686	0.092			0.000	0.000	1.927	0.054			0.001	0.000	2.043	0.041		
(Intercept)	3033	1.802	0.040	45.330	0.000	19.431	0.007	0.651	0.019	34.814	0.000	-1.872	0.000	1.415	0.029	48.571	0.000	33.124	0.012	1.497	0.061	24.714	0.000	3.609	0.002
Precipitation		0.000	0.000	4.636	0.000			0.000	0.000	0.357	0.721			0.000	0.000	5.942	0.000			0.000	0.000	2.369	0.018		
(Intercept)	3033	1.830	0.040	46.199	0.000	54.423	0.018	0.648	0.019	34.786	0.000	15.450	0.006	1.436	0.029	48.910	0.000	28.712	0.010	1.525	0.060	25.291	0.000	32.808	0.011
Latitude		-0.001	0.000	-7.544	0.000			0.000	0.000	4.182	0.000			0.000	0.000	-5.554	0.000			-0.001	0.000	-5.915	0.000		
(Intercept)	3033	1.813	0.039	45.931	0.000	34.910	0.012	0.653	0.019	35.089	0.000	1.759	0.001	1.426	0.029	48.916	0.000	41.111	0.014	1.505	0.060	25.023	0.000	17.511	0.006
Insularity [insular]		0.025	0.004	6.092	0.000			-0.005	0.002	-1.939	0.053			0.019	0.003	6.587	0.000			0.028	0.006	4.423	0.000		

Supplementary Table 3. Multi-predictor model of overall development period (n = 1665).

Term	Estimate	SE	T	P	ΔAIC	Partial R^2
(Intercept)	1.407	0.043	32.975	0.000	-	-
Adult body mass	0.136	0.007	19.248	0.000	336.182	0.184
Generation length	0.121	0.025	4.826	0.000	21.411	0.014
Clutch size	-0.071	0.016	-4.348	0.000	17.024	0.011
Parental care [uni]	-0.016	0.007	-2.356	0.019	3.607	0.003
Brood parasitism [parasitic]	-0.076	0.052	-1.464	0.143	0.168	0.001
Nest height	0.007	0.003	2.094	0.036	2.431	0.003
Forest dep [low]	0.002	0.005	0.404	0.686	-2.025	0.002
Forest dep [med]	-0.002	0.005	-0.316	0.752	-	-
Forest dep [high]	0.010	0.007	1.342	0.180	-	-
Diet [fruitnect]	0.011	0.009	1.222	0.222	3.865	0.007
Diet [invert]	-0.003	0.006	-0.563	0.573	-	-
Diet [plantseed]	0.013	0.007	1.801	0.072	-	-
Diet [vertfishscav]	0.019	0.009	2.067	0.039	-	-
Pelagic [pelagic]	0.042	0.015	2.923	0.004	6.627	0.005
Migration [migratory]	-0.012	0.004	-2.766	0.006	5.727	0.005
Temperature	0.000	0.001	-0.357	0.721	-1.871	0.000
Precipitation	0.000	0.000	-0.402	0.688	-1.837	0.000
Latitude	-0.001	0.000	-2.806	0.005	5.948	0.005
Insularity [insular]	0.030	0.007	4.392	0.000	17.407	0.012

Supplementary Table 4. Multi-predictor model of incubation fraction (n = 1685).

Term	Estimate	SE	T	P	ΔAIC	Partial R^2
(Intercept)	0.755	0.023	33.044	0.000	-	-
Adult body mass	-0.042	0.004	-9.774	0.000	91.678	0.054
Generation length	-0.019	0.015	-1.213	0.225	-0.518	0.001
Clutch size	-0.013	0.010	-1.325	0.185	-0.230	0.001
Parental care [uni]	0.010	0.004	2.447	0.015	4.025	0.004
Nest height	-0.004	0.002	-1.883	0.060	1.572	0.002
Diet [fruitnect]	-0.007	0.006	-1.151	0.250	13.605	0.013
Diet [invert]	0.010	0.004	2.742	0.006	-	-
Diet [plantseed]	-0.011	0.005	-2.221	0.026	-	-
Diet [vertfishscav]	0.002	0.006	0.388	0.698	-	-
Pelagic [pelagic]	0.002	0.010	0.180	0.857	-1.967	0.000
Nocturality [nocturnal]	0.064	0.022	2.916	0.004	6.554	0.005
Migration [migratory]	0.003	0.003	1.011	0.312	-0.969	0.001
Latitude	0.000	0.000	3.139	0.002	7.908	0.006

Supplementary Table 5. Multi-predictor model of overall development period using egg mass (n = 1448).

Term	Estimate	SE	T	P	ΔAIC	Partial R^2
(Intercept)	1.481	0.045	32.994	0.000	-	-
Egg mass	0.189	0.011	17.302	0.000	273.576	0.173
Generation length	0.114	0.027	4.236	0.000	16.079	0.012
Clutch size	-0.035	0.019	-1.855	0.064	1.483	0.002
Parental care [uni]	-0.016	0.007	-2.217	0.027	2.977	0.003
Brood parasitism [parasitic]	-0.069	0.054	-1.273	0.203	-0.357	0.001
Nest height	0.009	0.004	2.400	0.017	3.828	0.004
Forest dep [low]	0.001	0.005	0.195	0.845	-2.264	0.003
Forest dep [med]	-0.006	0.006	-0.908	0.364	-	-
Forest dep [high]	0.008	0.009	0.864	0.388	-	-
Diet [fruitnect]	0.015	0.010	1.455	0.146	14.221	0.015
Diet [invert]	-0.007	0.006	-1.137	0.256	-	-
Diet [plantseed]	0.021	0.008	2.601	0.009	-	-
Diet [vertfishscav]	0.024	0.010	2.407	0.016	-	-
Pelagic [pelagic]	0.045	0.016	2.831	0.005	6.103	0.006
Migration [migratory]	-0.014	0.005	-2.979	0.003	6.971	0.006
Temperature	0.000	0.001	-0.502	0.616	-1.744	0.000
Precipitation	0.000	0.000	-0.866	0.386	-1.239	0.001
Latitude	-0.001	0.000	-2.868	0.004	6.318	0.006
Insularity [insular]	0.032	0.008	3.923	0.000	13.526	0.011

Supplementary Table 6. Multi-predictor model of incubation fraction using egg mass (n = 1468).

Term	Estimate	SE	T	P	ΔAIC	Partial R^2
(Intercept)	0.723	0.024	29.898	0.000	-	-
Egg mass	-0.047	0.007	-7.051	0.000	47.362	0.033
Generation length	-0.025	0.017	-1.481	0.139	0.212	0.002
Clutch size	-0.023	0.012	-2.009	0.045	2.070	0.003
Parental care [uni]	0.010	0.005	2.229	0.026	3.006	0.003
Nest height	-0.006	0.002	-2.288	0.022	3.275	0.004
Diet [fruitnect]	-0.009	0.006	-1.356	0.175	15.138	0.016
Diet [invert]	0.011	0.004	2.750	0.006	-	-
Diet [plantseed]	-0.012	0.005	-2.236	0.026	-	-
Diet [vertfishscav]	0.001	0.006	0.227	0.820	-	-
Pelagic [pelagic]	0.004	0.010	0.357	0.721	-1.871	0.000
Nocturality [nocturnal]	0.069	0.024	2.831	0.005	6.068	0.005
Migration [migratory]	0.003	0.003	1.038	0.299	-0.913	0.001
Latitude	0.000	0.000	3.211	0.001	8.376	0.007

Supplementary Table 7. Multi-predictor model of incubation period (n = 1935).

Term	Estimate	SE	T	P	ΔAIC	Partial R^2
(Intercept)	1.186	0.030	39.999	0.000	-	-
Adult body mass	0.076	0.005	15.850	0.000	236.404	0.116
Generation length	0.078	0.017	4.658	0.000	19.793	0.011
Clutch size	-0.083	0.011	-7.799	0.000	58.467	0.031
Brood parasitism [parasitic]	-0.038	0.032	-1.197	0.231	-0.554	0.001
Nest height	0.000	0.002	0.030	0.976	-1.999	0.000
Forest dep [low]	0.001	0.003	0.166	0.868	0.181	0.003
Forest dep [med]	0.002	0.003	0.638	0.524	-	-
Forest dep [high]	0.011	0.005	2.266	0.024	-	-
Diet [fruitnect]	-0.004	0.006	-0.719	0.472	4.708	0.007
Diet [invert]	0.006	0.004	1.541	0.123	-	-
Diet [plantseed]	-0.004	0.005	-0.815	0.415	-	-
Diet [vertfishscav]	0.018	0.006	3.060	0.002	-	-
Pelagic [pelagic]	0.029	0.009	3.247	0.001	8.620	0.005
Nocturality [nocturnal]	0.104	0.030	3.412	0.001	9.722	0.006
Migration [migratory]	-0.007	0.003	-2.703	0.007	5.366	0.004
Precipitation	0.000	0.000	1.410	0.159	0.006	0.001
Latitude	0.000	0.000	-0.730	0.465	-1.462	0.000
Insularity [insular]	0.008	0.004	2.026	0.043	2.141	0.002

Supplementary Table 8. Multi-predictor model of fledging period (n = 1665).

Term	Estimate	SE	T	P	ΔAIC	Partial R^2
(Intercept)	1.057	0.070	15.073	0.000	-	-
Adult body mass	0.181	0.012	15.492	0.000	224.231	0.127
Generation length	0.121	0.041	2.920	0.004	6.586	0.005
Clutch size	-0.046	0.027	-1.720	0.086	0.983	0.002
Parental care [uni]	-0.026	0.011	-2.424	0.015	3.924	0.004
Nest height	0.012	0.005	2.289	0.022	3.283	0.003
Diet [fruitnect]	0.018	0.015	1.191	0.234	4.125	0.007
Diet [invert]	-0.016	0.009	-1.748	0.081	-	-
Diet [plantseed]	0.021	0.012	1.692	0.091	-	-
Diet [vertfishscav]	0.007	0.015	0.442	0.658	-	-
Pelagic [pelagic]	0.033	0.024	1.363	0.173	-0.125	0.001
Migration [migratory]	-0.014	0.007	-1.853	0.064	1.462	0.002
Temperature	-0.001	0.001	-0.858	0.391	-1.257	0.000
Precipitation	0.000	0.000	-0.932	0.352	-1.124	0.001
Latitude	-0.001	0.000	-3.002	0.003	7.072	0.005
Insularity [insular]	0.044	0.011	3.903	0.000	13.310	0.009

Supplementary Table 9. Multi-predictor model of incubation period using egg mass (n = 1673).

Term	Estimate	SE	T	P	ΔAIC	Partial R^2
(Intercept)	1.202	0.028	42.241	0.000	-	-
Egg mass	0.120	0.007	17.125	0.000	271.076	0.151
Generation length	0.069	0.017	4.085	0.000	14.791	0.010
Clutch size	-0.063	0.011	-5.465	0.000	27.942	0.018
Brood parasitism [parasitic]	-0.036	0.031	-1.165	0.244	-0.628	0.001
Nest height	0.001	0.002	0.460	0.645	-1.786	0.000
Forest dep [low]	0.001	0.003	0.380	0.704	-2.352	0.002
Forest dep [med]	-0.002	0.004	-0.588	0.557	-	-
Forest dep [high]	0.006	0.005	1.135	0.256	-	-
Diet [fruitnect]	-0.006	0.006	-0.961	0.336	6.923	0.009
Diet [invert]	0.005	0.004	1.349	0.178	-	-
Diet [plantseed]	0.001	0.005	0.151	0.880	-	-
Diet [vertfishscav]	0.021	0.006	3.585	0.000	-	-
Pelagic [pelagic]	0.034	0.009	3.813	0.000	12.644	0.009
Nocturality [nocturnal]	0.091	0.030	3.013	0.003	7.158	0.005
Migration [migratory]	-0.009	0.003	-3.043	0.002	7.338	0.006
Precipitation	0.000	0.000	1.536	0.125	0.385	0.001
Latitude	0.000	0.000	-0.501	0.617	-1.746	0.000
Insularity [insular]	0.012	0.005	2.601	0.009	4.829	0.004

Supplementary Table 10. Multi-predictor model of fledging period using egg mass (n = 1448).

Term	Estimate	SE	T	P	ΔAIC	Partial R^2
(Intercept)	1.171	0.076	15.477	0.000	-	-
Egg mass	0.237	0.018	12.837	0.000	155.721	0.103
Generation length	0.124	0.046	2.724	0.007	5.483	0.005
Clutch size	0.003	0.032	0.086	0.931	-1.992	0.000
Parental care [uni]	-0.028	0.012	-2.267	0.024	3.186	0.004
Nest height	0.016	0.006	2.479	0.013	4.201	0.004
Diet [fruitnect]	0.024	0.017	1.396	0.163	10.808	0.013
Diet [invert]	-0.021	0.010	-2.054	0.040	-	-
Diet [plantseed]	0.031	0.014	2.260	0.024	-	-
Diet [vertfishscav]	0.010	0.017	0.610	0.542	-	-
Pelagic [pelagic]	0.032	0.027	1.214	0.225	-0.512	0.001
Migration [migratory]	-0.016	0.008	-2.033	0.042	2.173	0.003
Temperature	-0.001	0.001	-1.013	0.311	-0.963	0.001
Precipitation	0.000	0.000	-1.432	0.152	0.072	0.001
Latitude	-0.001	0.000	-3.109	0.002	7.743	0.007
Insularity [insular]	0.044	0.014	3.200	0.001	8.315	0.007

Supplementary Table 11. Phylogenetic covariance model comparison results.

		Development period				Incubation fraction				Incubation period				Fledging period			
**Predictor(s)**	**Covariance model**	**logLik**	**AIC**	**ΔAIC**	**AICw**	**logLik**	**AIC**	**ΔAIC**	**AICw**	**logLik**	**AIC**	**ΔAIC**	**AICw**	**logLik**	**AIC**	**ΔAIC**	**AICw**
Adult body mass	BM	1486.75	-2967.50	5116.74	0.00	3873.01	-7740.03	3276.01	0.00	2590.49	-5174.99	4750.11	0.00	421.96	-837.92	4346.51	0.00
	OU	2133.99	-4259.97	3824.27	0.00	4492.24	-8976.49	2039.56	0.00	3106.38	-6204.76	3720.33	0.00	1013.86	-2019.72	3164.71	0.00
	Lambda	4046.12	-8084.24	0.00	1.00	5512.02	-11016.04	0.00	1.00	4966.55	-9925.10	0.00	1.00	2596.22	-5184.43	0.00	1.00
Egg mass	BM	2162.32	-4318.64	2385.34	0.00	3467.41	-6928.82	2140.53	0.00	3349.50	-6693.00	2003.27	0.00	1057.50	-2109.01	1998.46	0.00
	OU	2448.18	-4888.35	1815.63	0.00	3853.68	-7699.35	1370.00	0.00	3551.89	-7095.79	1600.48	0.00	1297.59	-2587.19	1520.28	0.00
	Lambda	3355.99	-6703.98	0.00	1.00	4538.67	-9069.35	0.00	1.00	4352.13	-8696.27	0.00	1.00	2057.73	-4107.47	0.00	1.00
Generation length	BM	1445.14	-2884.29	3998.35	0.00	3511.84	-7017.68	2977.17	0.00	2729.55	-5453.10	3318.42	0.00	386.65	-767.29	3592.50	0.00
	OU	1799.32	-3590.63	3292.01	0.00	4063.55	-8119.11	1875.74	0.00	2941.66	-5875.32	2896.21	0.00	779.27	-1550.54	2809.25	0.00
	Lambda	3445.32	-6882.64	0.00	1.00	5001.43	-9994.85	0.00	1.00	4389.76	-8771.52	0.00	1.00	2183.90	-4359.79	0.00	1.00
Clutch size	BM	1138.44	-2270.87	4921.89	0.00	3664.95	-7323.90	3077.46	0.00	2128.48	-4250.97	5000.18	0.00	198.66	-391.33	4177.25	0.00
	OU	1533.02	-3058.03	4134.73	0.00	4170.52	-8333.04	2068.33	0.00	2484.65	-4961.29	4289.86	0.00	587.69	-1167.38	3401.20	0.00
	Lambda	3600.38	-7192.76	0.00	1.00	5204.68	-10401.36	0.00	1.00	4629.57	-9251.15	0.00	1.00	2288.29	-4568.58	0.00	1.00
Developmental mode	BM	1424.88	-2841.76	1019.50	0.00	2434.45	-4860.90	1085.30	0.00	2047.54	-4087.08	1332.28	0.00	732.67	-1457.34	808.57	0.00
	OU	1513.81	-3017.63	843.62	0.00	2639.19	-5268.38	677.82	0.00	2123.38	-4236.77	1182.59	0.00	828.46	-1646.92	618.99	0.00
	Lambda	1935.63	-3861.25	0.00	1.00	2978.10	-5946.20	0.00	1.00	2714.68	-5419.36	0.00	1.00	1137.95	-2265.91	0.00	1.00
Parental care	BM	2100.33	-4194.65	2007.57	0.00	3571.58	-7137.15	2077.03	0.00	3282.88	-6559.76	1421.61	0.00	1049.99	-2093.98	1899.73	0.00
	OU	2267.69	-4527.38	1674.85	0.00	3950.07	-7892.13	1322.05	0.00	3380.97	-6753.94	1227.43	0.00	1271.90	-2535.79	1457.91	0.00
	Lambda	3105.11	-6202.22	0.00	1.00	4611.09	-9214.18	0.00	1.00	3994.68	-7981.37	0.00	1.00	2000.85	-3993.70	0.00	1.00
Brood parasitism	BM	1137.09	-2268.19	5076.23	0.00	3830.42	-7654.84	3193.21	0.00	2138.93	-4271.87	5110.48	0.00	167.74	-329.48	4382.28	0.00
	OU	1616.97	-3225.94	4118.47	0.00	4369.53	-8731.07	2116.98	0.00	2589.48	-5170.96	4211.39	0.00	641.32	-1274.64	3437.12	0.00
	Lambda	3676.21	-7344.42	0.00	1.00	5428.02	-10848.05	0.00	1.00	4695.17	-9382.35	0.00	1.00	2359.88	-4711.76	0.00	1.00
Nest height	BM	992.26	-1978.52	2853.92	0.00	2601.97	-5197.94	2094.98	0.00	1894.01	-3782.01	2683.44	0.00	182.98	-359.96	2538.52	0.00
	OU	1279.30	-2550.60	2281.84	0.00	2985.65	-5963.30	1329.62	0.00	2126.51	-4245.01	2220.44	0.00	478.01	-948.02	1950.47	0.00
	Lambda	2420.22	-4832.44	0.00	1.00	3650.46	-7292.92	0.00	1.00	3236.73	-6465.45	0.00	1.00	1453.24	-2898.49	0.00	1.00
Nest type	BM	1213.51	-2417.02	3666.31	0.00	3556.60	-7103.19	1749.03	0.00	1916.87	-3823.74	3647.59	0.00	511.98	-1013.95	2911.85	0.00
	OU	1524.57	-3037.13	3046.20	0.00	3825.88	-7639.77	1212.46	0.00	2222.41	-4432.81	3038.52	0.00	783.52	-1555.03	2370.77	0.00
	Lambda	3047.66	-6083.33	0.00	1.00	4432.11	-8852.23	0.00	1.00	3741.67	-7471.33	0.00	1.00	1968.90	-3925.80	0.00	1.00
Forest dependancy	BM	1432.99	-2855.97	4397.74	0.00	3815.15	-7620.29	3096.81	0.00	2658.16	-5306.33	3966.68	0.00	374.54	-739.08	3912.45	0.00
	OU	1817.11	-3622.22	3631.49	0.00	4324.62	-8637.23	2079.87	0.00	2970.15	-5928.30	3344.71	0.00	770.09	-1528.17	3123.36	0.00
	Lambda	3632.86	-7253.71	0.00	1.00	5364.55	-10717.11	0.00	1.00	4642.51	-9273.01	0.00	1.00	2331.76	-4651.53	0.00	1.00
Diet	BM	1153.91	-2295.83	5093.11	0.00	3838.21	-7664.42	3217.56	0.00	2161.57	-4311.15	5087.50	0.00	180.73	-349.46	4405.14	0.00
	OU	1644.71	-3275.42	4113.52	0.00	4393.13	-8772.26	2109.71	0.00	2616.44	-5218.89	4179.76	0.00	667.64	-1321.28	3433.32	0.00
	Lambda	3701.47	-7388.94	0.00	1.00	5447.99	-10881.98	0.00	1.00	4706.33	-9398.65	0.00	1.00	2384.30	-4754.60	0.00	1.00
Pelagic	BM	1137.69	-2269.37	5119.40	0.00	3838.73	-7671.46	3181.98	0.00	2141.33	-4276.66	5144.04	0.00	169.15	-332.31	4405.34	0.00
	OU	1618.96	-3229.92	4158.86	0.00	4379.91	-8751.83	2101.62	0.00	2584.40	-5160.80	4259.90	0.00	646.12	-1284.24	3453.41	0.00
	Lambda	3698.39	-7388.78	0.00	1.00	5430.72	-10853.44	0.00	1.00	4714.35	-9420.69	0.00	1.00	2372.82	-4737.65	0.00	1.00
Nocturnality	BM	1137.06	-2268.12	5071.99	0.00	3830.97	-7655.93	3201.95	0.00	2139.40	-4272.80	5113.19	0.00	167.78	-329.57	4381.44	0.00
	OU	1614.55	-3221.09	4119.02	0.00	4371.64	-8735.29	2122.59	0.00	2588.73	-5169.45	4216.53	0.00	639.88	-1271.76	3439.25	0.00
	Lambda	3674.06	-7340.12	0.00	1.00	5432.94	-10857.88	0.00	1.00	4696.99	-9385.98	0.00	1.00	2359.51	-4711.01	0.00	1.00
Migration	BM	1135.09	-2264.19	5051.21	0.00	3786.51	-7567.01	3161.43	0.00	2132.33	-4258.66	5055.44	0.00	186.56	-367.12	4322.33	0.00
	OU	1599.77	-3191.53	4123.86	0.00	4318.86	-8629.73	2098.72	0.00	2562.82	-5117.64	4196.45	0.00	645.27	-1282.54	3406.91	0.00
	Lambda	3661.70	-7315.39	0.00	1.00	5368.22	-10728.44	0.00	1.00	4661.05	-9314.10	0.00	1.00	2348.72	-4689.45	0.00	1.00
Temperature	BM	1082.56	-2159.13	5003.75	0.00	3758.71	-7511.41	3080.82	0.00	2055.13	-4104.26	5073.45	0.00	149.33	-292.67	4279.59	0.00
	OU	1555.81	-3103.61	4059.27	0.00	4275.90	-8543.79	2048.44	0.00	2499.50	-4991.01	4186.71	0.00	613.08	-1218.16	3354.10	0.00
	Lambda	3585.44	-7162.88	0.00	1.00	5300.12	-10592.23	0.00	1.00	4592.86	-9177.72	0.00	1.00	2290.13	-4572.25	0.00	1.00
Precipitation	BM	1130.00	-2254.01	4920.87	0.00	3727.24	-7448.47	3141.05	0.00	2126.30	-4246.60	4962.45	0.00	176.91	-347.81	4225.88	0.00
	OU	1584.33	-3160.65	4014.22	0.00	4259.90	-8511.81	2077.71	0.00	2544.28	-5080.55	4128.49	0.00	628.50	-1248.99	3324.70	0.00
	Lambda	3591.44	-7174.88	0.00	1.00	5298.76	-10589.52	0.00	1.00	4608.52	-9209.04	0.00	1.00	2290.85	-4573.69	0.00	1.00
Latitude	BM	1256.06	-2506.12	4703.71	0.00	3799.97	-7593.93	3012.91	0.00	2211.20	-4416.40	4788.28	0.00	314.15	-622.30	3980.58	0.00
	OU	1671.00	-3334.01	3875.83	0.00	4289.78	-8571.56	2035.28	0.00	2610.31	-5212.62	3992.06	0.00	715.42	-1422.84	3180.03	0.00
	Lambda	3608.92	-7209.83	0.00	1.00	5307.42	-10606.84	0.00	1.00	4606.34	-9204.68	0.00	1.00	2305.44	-4602.87	0.00	1.00
Insularity	BM	1026.14	-2046.29	5041.01	0.00	3642.89	-7279.78	3165.46	0.00	1999.47	-3992.95	5084.95	0.00	88.90	-171.79	4339.00	0.00
	OU	1501.49	-2994.98	4092.32	0.00	4179.10	-8350.19	2095.05	0.00	2444.99	-4881.98	4195.92	0.00	559.42	-1110.84	3399.95	0.00
	Lambda	3547.65	-7087.30	0.00	1.00	5226.62	-10445.24	0.00	1.00	4542.95	-9077.90	0.00	1.00	2259.39	-4510.79	0.00	1.00
Multipredictor model (with adult body mass)	BM	1574.27	-3106.54	1056.80	0.00	2263.11	-4496.22	1196.31	0.00	2131.59	-4223.18	2030.19	0.00	753.29	-1472.57	1002.11	0.00
	OU	1729.57	-3415.14	748.21	0.00	2558.84	-5085.69	606.84	0.00	2287.10	-4532.20	1721.17	0.00	959.60	-1883.20	591.49	0.00
	Lambda	2103.67	-4163.35	0.00	1.00	2862.26	-5692.53	0.00	1.00	3147.68	-6253.37	0.00	1.00	1255.34	-2474.69	0.00	1.00
Multipredictor model (with egg mass)	BM	1871.56	-3703.12	1435.45	0.00	2678.98	-5329.96	1684.83	0.00	3172.19	-6306.37	1527.61	0.00	807.77	-1583.54	1521.54	0.00
	OU	2054.08	-4066.16	1072.41	0.00	3039.69	-6049.38	965.42	0.00	3298.38	-6556.77	1277.22	0.00	1066.99	-2099.99	1005.10	0.00
	Lambda	2590.28	-5138.57	0.00	1.00	3522.40	-7014.79	0.00	1.00	3936.99	-7833.99	0.00	1.00	1569.54	-3105.09	0.00	1.00

Supplementary References

1. V. Hamburger, H. L. Hamilton, A series of normal stages in the development of the chick embryo. *J. Morphol.* **88**, 49-92 (1951).
2. H. Wilman *et al.*, EltonTraits 1.0: species-level foraging attributes of the world's birds and mammals. *Ecology* **95**, 2027 (2014).
3. N. P. Myhrvold *et al.*, An amniote life-history database to perform comparative analyses with birds, mammals, and reptiles. *Ecology* **96**, 3109 (2015).
4. J. del Hoyo, A. Elliott, J. Sargatal, D. A. Christe, *The Handbook of the Birds of the World, vols 1-16* (Lynx Edicions, Barcelona, 1992–2011).
5. J. M. Starck, "Evolution of avian ontogenies" in Current Ornithology, D. M. Power, Ed. (Plenum Press, New York, 1993), pp. 275-366.
6. C. R. Cooney, H. E. A. MacGregor, N. Seddon, J. A. Tobias, Multi-modal signal evolution in birds: re-assessing a standard proxy for sexual selection. *Proc. R. Soc. London Ser. B* **285**, 20181557 (2018).
7. W. Jetz, C. H. Sekercioglu, K. Böhning-Gaese, The worldwide variation in avian clutch size across species and space. *PLoS Biol.* **6**, e303 (2008).
8. S. E. Fick, R. J. Hijmans, WorldClim 2: new 1-km spatial resolution climate surfaces for global land areas. *International Journal of Climatology* **37**, 4302-4315 (2017).
9. P. Weigelt, W. Jetz, H. Kreft, Bioclimatic and physical characterization of the world's islands. *Proc. Natl. Acad. Sci. U.S.A.* **110**, 15307-15312 (2013).
10. J. C. Daniel, An embryological comparison of the domestic fowl and the red-winged blackbird. *Auk* **74**, 340-358 (1957).
11. R. A. Montgomery, C. J. Burke, S. M. Byers, A field guide to the aging of wood duck embryos. *J. Wildl. Manage.* **42**, 432-437 (1978).
12. H. U. Koecke, Normalstadien der embryonal-entwicklung bei der hausente (*Anas boschas domestica*). *Embryologia* **4**, 55-78 (1958).
13. J. A. Cooper, B. D. J. Batt, Criteria for aging giant canada goose embryos. *J. Wildl. Manage.* **36**, 1267-1270 (1972).
14. A. G. Hendrickx, R. Hanzlik, Developmental stages of the bob-white quail embryo (*Colinus virginianus*). *Biological Bulletin* **129**, 523-531 (1965).
15. G. B. Olea, M. T. Sandoval, Embryonic development of *Columba livia* (Aves: Columbiformes) from an altricial-precocial perspective. *Revista Colombiana de Ciencias Pecuarias* **25**, 3-13 (2012).
16. S. J. Ainsworth, R. L. Stanley, D. J. Evans, Developmental stages of the Japanese quail. *J. Anat.* **216**, 3-15 (2010).
17. H. Nagai *et al.*, Embryonic development of the emu, *Dromaius novaehollandiae*. *Dev. Dyn.* **240**, 162-175 (2011).
18. J. M. Pisenti, G. M. Santolo, J. T. Yamamoto, A. A. Morzenti, Embryonic development of the american kestrel (*Falco sparverius*): external criteria for staging. *Journal of Raptor Research* **35**, 194-206 (2001).
19. M. Yamasaki, A. Tonosaki, Developmental stages of the society finch, *Lonchura striata var. domestica*. *Dev. Growth Differ.* **30**, 515-542 (1988).
20. A. M. Mun, I. L. Kosin, Developmental stages of the broad breasted bronze turkey embryo. *Biological Bulletin* **119**, 90-97 (1960).
21. A. Ancel, S. Liess, H. Girard, Embryonic development of the domestic guinea fowl (*Numida meleagris*). *J. Zool.* **235**, 621-634 (1995).
22. N. Hemmings, T. R. Birkhead, Consistency of passerine embryo development and the use of embryonic staging in studies of hatching failure. *Ibis* **158**, 43-50 (2016).
23. D. C. Powell, R. J. Aulerich, R. J. Balander, K. L. Stromborg, S. J. Bursian, A photographic guide to the development of double-crested cormorant embryos. *Colonial Waterbirds* **21**, 348-355 (1998).
24. R. J. Fant, Criteria for aging pheasant embryos. *J. Wildl. Manage.* **21**, 324-328 (1957).

25. C. Herbert, A timed series of embryonic developmental stages of the Adelie penguin (*Pygoscelis adeliae*) from Signy Island, South Orkney Islands. *British Antarctic Survey Bulletin* **14**, 45-67 (1967).
26. C. Köppl, E. Futterer, B. Nieder, R. Sistermann, H. Wagner, Embryonic and posthatching development of the barn owl (*Tyto alba*): reference data for age determination. *Dev. Dyn.* **233**, 1248-1260 (2005).
