# Supplementary material for: Ecology and allometry predict the evolution of avian developmental durations
Source: Nat Commun. 2020 May 14;11:2383. doi: 10.1038/s41467-020-16257-x (PMC7224302; doi:10.1038/s41467-020-16257-x)
Supplement: Supplementary file 2 — Reporting Summary [file 41467_2020_16257_MOESM2_ESM.pdf]

nature research

Corresponding author(s): Christopher R. Cooney

Last updated by author(s): Apr 16, 2020

Reporting Summary

Nature Research wishes to improve the reproducibility of the work that we publish. This form provides structure for consistency and transparency in reporting. For further information on Nature Research policies, see Authors & Referees and the Editorial Policy Checklist .

Statistics

For all statistical analyses, confirm that the following items are present in the figure legend, table legend, main text, or Methods section.

n/a	Confirmed	
☐	☒	The exact sample size (*n*) for each experimental group/condition, given as a discrete number and unit of measurement
☐	☒	A statement on whether measurements were taken from distinct samples or whether the same sample was measured repeatedly
☐	☒	The statistical test(s) used AND whether they are one- or two-sided<br>*Only common tests should be described solely by name; describe more complex techniques in the Methods section.*
☐	☒	A description of all covariates tested
☐	☒	A description of any assumptions or corrections, such as tests of normality and adjustment for multiple comparisons
☐	☒	A full description of the statistical parameters including central tendency (e.g. means) or other basic estimates (e.g. regression coefficient) AND variation (e.g. standard deviation) or associated estimates of uncertainty (e.g. confidence intervals)
☐	☒	For null hypothesis testing, the test statistic (e.g. *F*, *t*, *r*) with confidence intervals, effect sizes, degrees of freedom and *P* value noted<br>*Give P values as exact values whenever suitable.*
☒	☐	For Bayesian analysis, information on the choice of priors and Markov chain Monte Carlo settings
☒	☐	For hierarchical and complex designs, identification of the appropriate level for tests and full reporting of outcomes
☐	☒	Estimates of effect sizes (e.g. Cohen's *d*, Pearson's *r*), indicating how they were calculated

Our web collection on statistics for biologists contains articles on many of the points above.

Software and code

Policy information about availability of computer code

Data collection	No software used.
Data analysis	Data analysed in R v3.5.0. All analyses used base R functions or code provided in previously published R packages. Code used to run our analyses is available at https://github.com/christophercooney/Avian-developmental-durations.

For manuscripts utilizing custom algorithms or software that are central to the research but not yet described in published literature, software must be made available to editors/reviewers. We strongly encourage code deposition in a community repository (e.g. GitHub). See the Nature Research guidelines for submitting code & software for further information.

Data

Policy information about availability of data

All manuscripts must include a data availability statement. This statement should provide the following information, where applicable:

- Accession codes, unique identifiers, or web links for publicly available datasets
- A list of figures that have associated raw data
- A description of any restrictions on data availability

All data analysed in this study is provided as part of the Source Data file accompanying the manuscript.

Field-specific reporting

Please select the one below that is the best fit for your research. If you are not sure, read the appropriate sections before making your selection.

☐ Life sciences ☐ Behavioural & social sciences ☒ Ecological, evolutionary & environmental sciences

Ecological, evolutionary & environmental sciences study design

All studies must disclose on these points even when the disclosure is negative.

Study description	This study analyses data from over 3000 bird species in a phylogenetic comparative framework to test hypotheses for factors influencing variation in developmental durations across species.
Research sample	This research was focused on birds and the research sample consisted of the total number of species for which we could find published information regarding developmental durations (3096 species).
Sampling strategy	Sampling was dictated by the availability of published information regarding developmental durations.
Data collection	We recorded information from published sources (e.g. journal articles, handbooks, published compilations etc.) regarding the length of developmental periods for as many bird species as possible. We also collected a more detailed dataset of embryonic developmental time points for 20 species from published developmental studies. We combined this a mix of previously published and newly collected data describing interspecific variation in species' life history, ecological and geographic traits. Published data were extracted from the relevant repositories and newly collected data were assembled in a similar way to developmental duration data (i.e. collated from literature searches and published sources). Full details are provided in the main text.
Timing and spatial scale	Data were collected from the literature over the period of several years for a global sample of bird species.
Data exclusions	To improve data quality we removed clear outliers that must reflect measurement error (i.e. incubation lengths < 8 or > 90 days; n = 6). This is stated in the main text.
Reproducibility	N/A - study is not experimental.
Randomization	N/A - data were collected for as many extant bird species as possible, with final sample size dictated by data availability.
Blinding	N/A - data were collected for as many extant bird species as possible, with final sample size dictated by data availability.

Did the study involve field work? ☐ Yes ☒ No

Reporting for specific materials, systems and methods

We require information from authors about some types of materials, experimental systems and methods used in many studies. Here, indicate whether each material, system or method listed is relevant to your study. If you are not sure if a list item applies to your research, read the appropriate section before selecting a response.

Materials & experimental systems

n/a	Involved in the study	
☒	☐	Antibodies
☒	☐	Eukaryotic cell lines
☒	☐	Palaeontology
☒	☐	Animals and other organisms
☒	☐	Human research participants
☒	☐	Clinical data

Methods

n/a	Involved in the study	
☒	☐	ChIP-seq
☒	☐	Flow cytometry
☒	☐	MRI-based neuroimaging
